# ﻿Three new *Cerodontha* species from Inner Mongolia and Qinghai with a checklist of thirty-two Chinese species (Diptera, Agromyzidae)

**DOI:** 10.3897/zookeys.1266.177044

**Published:** 2026-01-05

**Authors:** Xin-Ting Fu, Li Shi, Shi-Mingzhe Wang

**Affiliations:** 1 College of Horticulture and Plant Protection, Inner Mongolia Agricultural University, Hohhot, Inner Mongolia, 010018, China Inner Mongolia Agricultural University Hohhot China; 2 College of Food Science and Engineering, Inner Mongolia Agricultural University, Hohhot, Inner Mongolia, 010018, China Inner Mongolia Agricultural University Hohhot China

**Keywords:** Agromyzidae, morphological structure, new record, northern China, Phytomyzinae

## Abstract

Three species, Cerodontha (Dizygomyza) flavilunulata**sp. nov.**, C. (D.) granditerga**sp. nov.**, C. (D.) tumefacta**sp. nov.**, are new to science, and four species, C. (Cerodontha) flavicornis (Egger, 1862), C. (D.) labradorensis Spencer, 1969, C. (Poemyza) beigerae Nowakowski, 1972, and C. (P.) muscina (Meigen, 1830), are recorded from China for the first time. A key and a checklist with photographs of morphological features and male genitalia are presented.

## ﻿Introduction

The genus *Cerodontha* Rondani, 1861 is different from other genera of Agromyzidae in the following features: first flagellomere with short spine or projection at anterodistal corner in subgenus Cerodontha, or in male normally greatly enlarged in subgenus Dizygomyza (Spencer 1987); lunule higher than wide, often convex, conspicuously produced into emarginate frons; mesonotum with a pair of prescutellar setae often absent, but present in subgenera *Butomomyza* and *Dizygomyza*; scutellum with lateral and apical scutellar setae often present, but absent in subgenus Cerodontha and *Xenophytomyza*; wing with costa extending to M_1_; epandrium with a pair of long or short claviform processes produced from the ventral side of subepandrial sclerite in posterior view; distiphallus with two long or short tubules, swollen or slightly separated at apex, sometimes forming small bell-like funnel (Lonsdale and von Tschirnhaus 2021); oviscape black, setigerous (Guglya 2021).

Seven valid subgenera of *Cerodontha* are recognized globally (Nowakowski 1962, 1967; Boucher 2012; Lonsdale and von Tschirnhaus 2021): subgenus Cerodontha sensu stricto (62 species), subgenus ButomomyzaNowakowski 1967 (37 species), subgenus Dizygomyza (Hendel 1920) (71 species); subgenus Icteromyza (Hendel 1931) (31 species); subgenus Phytagromyza (Hendel 1920) (3 species); subgenus Poemyza (Hendel 1931) (84 species); and subgenus Xenophytomyza (Frey 1946) (10 species). All of seven subgenera occur both in the Palaearctic and Nearctic regions, and five subgenera *Butomomyza*, *Cerodontha*, *Dizygomyza*, *Icteromyza* and *Poemyza* in the Oriental, Afrotropical, Australasian, and Oceanian regions, and four subgenera *Butomomyza*, *Cerodontha*, *Dizygomyza* and *Icteromyza* in the Neotropical region. Zlobin (1979, 1984a, 1986, 1993a, 1993b, 1993c, 1996, 2000) reviewed five subgenera *Cerodontha*, *Dizygomyza*, *Icteromyza*, *Poemyza* and *Xenophytomyza* in the Palaearctic region. Boucher (2002, 2003, 2005, 2008, 2012) systematically revised four subgenera *Cerodontha*, *Xenophytomyza*, *Dizygomyza* and *Icteromyza* in the Nearctic and Neotropical regions. The keys to subgenera were provided by Spencer and Steyskal (1986), Boucher (2010), and Lonsdale and von Tschirnhaus (2021).

Based on the literature review and two databases (von Tschirnhaus and Groll 2024a, 2024b; Bánki et al. 2025), the genus *Cerodontha* Rondani, 1861 (Diptera: Agromyzidae: Phytomyzinae) is a worldwide genus with 298 species distributed among seven subgenera (Nowakowski 1967, 1972; Spencer 1969) as of July 2025, with 140 species in the Palaearctic region, 49 species in the Oriental region, 25 species in China (Sasakawa 2006; Guglya 2021; Lonsdale and von Tschirnhaus 2021; de Sousa et al. 2024; Boucher and Pollet 2025), and no species reported from Inner Mongolia and Qinghai province before this research.

**Figure 1.**
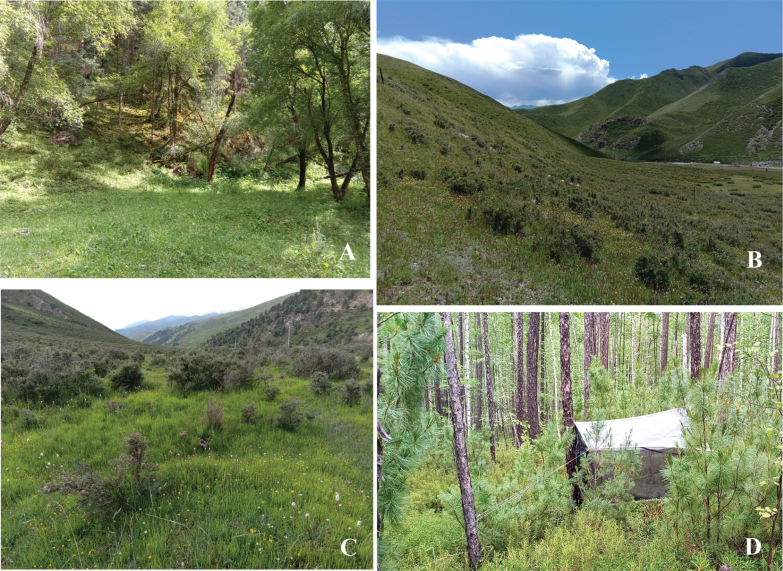
Field observation of three new species. Habitat of C. (D.) granditerga sp. nov. from **A** and **C**, Cerodontha (Dizygomyza) flavilunulata sp. nov. from **D**, and C. (D.) tumefacta sp. nov. from **B** and **C**. The photograph taken from: **A.** China, Qinghai Province, Menyuan County, Zhugu Town, Sigou, 28.VII.2021; **B.** China, Qinghai Province, Qilian County, Ebu Town, the southern slope of Longkongdaban, 31.VII.2021; **C.** China, Qinghai Province, Qilian County, Lujiaogou, 01.VIII.2021; **D.** China, Inner Mongolia, Genhe City, Mangui Town, the northern primitive forest region of Greater Khingan Mountains, Aba River First Branch Line, 04.VII.2022.

All examined specimens were collected by the sweep net and malaise trap from the northern primitive forest region in the Greater Khingan Mountains of Inner Mongolia and Qilian Mountain National Park (Qinghai Section; habitats photos of insect collection, Fig. 1A–D). The general descriptions of the two regions are as follows: the northern primitive forest region in the Greater Khingan Mountains of Inner Mongolia is situated at the southern boundary of the Eurasian permafrost zone (Wei 2020), and this area serves as a natural ecological barrier for the Songnen Plain, Hulunbeir Grassland as well as being the major grain-producing regions in northeast China, playing a crucial and irreplaceable role in water conservation, soil protection, carbon sequestration, oxygen release, environmental purification, biodiversity preservation, greenhouse gas reduction, and climate change mitigation (Sheng 2023). Its significance extends not only to the protection of vegetation and rare wildlife in the Greater Khingan Mountains but also to its high scientific research value (Ma 2021). The Qilian Mountain National Nature Reserve in Qinghai Province is located in the northeastern part of Qinghai Province, on the edge of the Qinghai-Tibet Plateau, and it boasts a unique plateau ecosystem, with a large number of wetlands, glaciers, and rare and endangered wild animals and plants, and it serves as a world-class high-altitude germplasm resource bank and a wildlife migration corridor, holding extremely high scientific research value (Yang 2019).

In this paper, three new species of the subgenus Dizygomyza are described and four described species are recorded for the first time in China. A key to all 32 species of the genus *Cerodontha* in China and a checklist of these species are provided.

## ﻿Materials and methods

The general terminology follows Lonsdale (2021) and Guglya (2021). To prepare the genitalia, the apex of the abdomen was excised and subjected to maceration in warm lactic acid for 20–25 minutes. Following this treatment, specimens were rinsed with purified water to facilitate dissection and morphological analysis. After examination in glycerin, the genital structures were transferred and stored in a microvial containing glycerin for long-term preservation.

Specimens were examined with a Nikon 1270 dissection microscope. Images of adult individuals and genitalia were captured with a DMC6200 digital camera mounted on a Leica M205FA dissecting microscope. Sequential images were subsequently combined into composite montages using Leica Application Suite X 5.0.2.24429 software. Final image processing, including optimization and editing, was performed using Adobe Photoshop CS 6.0® to enhance clarity and consistency.

The type specimens of the new species and other examined specimens are deposited in the Insect Collection of Inner Mongolia Agricultural University, Hohhot, Inner Mongolia, China (IMAU). Two new records for Inner Mongolia and Qinghai are marked with an asterisk (*) and four new records for China are marked with a hash (^#^) in the distribution sections.

Abbreviations of morphological terms used in the text: ***acr***—acrostichal setulae, ***dc***—dorsocentral seta, ***ori***—inferior fronto-orbital seta, ***ors***—superior fronto-orbital seta, **M_1_**—first branch of vein Media, **M_4_**—fourth branch of vein Media, ***r-m***—crossvein radial-medial.

## ﻿Taxonomy

**Subgenus Cerodontha Rondani, 1861**

### ﻿ Cerodontha (Cerodontha) flavicornis

Taxon classificationAnimaliaDipteraAgromyzidae

(Egger, 1862)

90CBF473-60B0-5890-9A91-92E056875D5B

Figs 2A–N, 3A–L

Ceratomyza flavicornis Egger, 1862: 782; Schiner 1864: 311.
Cerodontha flavicornis : Hendel 1920: 169; Hering 1927: 160; Stackelberg 1933: 465.
Cerodontha (Cerodontha) flavicornis : Nowakowski 1967: 656; 1972: 738; 1973: 51; Rohdendorf 1970: 270; Zlobin 1979: 874; Soós and Papp 1984: 286; Spasić 1996: 114; Černý 2018: 125; Černý and Bächli 2018: 134.

#### Specimens examined.

China, Qinghai Province: • 4♂♂15♀♀ (IMAU), Delingha City, entrance of Cypress Mountain Forest Park, 3296 m, 37°26'58.01"N, 97°20'13.54"E, 09.VIII.2021, leg. Li Shi; • 3♀♀ (IMAU), Haidong City, Huzhu County, Bazha Town, Baimuxia Village, 3070 m, 37°00'09.38"N, 102°07'36.04"E, 24.VII.2021, leg. Li Shi; • 2♂♂20♀♀ (IMAU), Tianjun County, grassland near Guanjiaoshan Highway, 3626.6 m, 37°9'32.09"N, 98°51'34.81"E, 08.VIII.2021, leg. Li Shi; • 2♀♀ (IMAU), Menyuan County, Xianmi Town, Meihua Village, 3148 m, 37°17'12.17"N, 102°08'45.34"E, 30.VII.2021, leg. Li Shi; • 8♂♂7♀♀ (IMAU), Menyuan County, Xianmi Town, Qiaotan Village, Qihankaigou, 2715 m, 37°09'33.51"N, 102°02'05.13"E, 30.VII.2021, leg. Li Shi; • 10♂♂5♀♀ (IMAU), Menyuan County, Zhugu Town, Dezong Village, 2894 m, 37°13'02.69"N, 102°11'58.20"E, 26.VII.2021, leg. Li Shi; • 38♂♂63♀♀ (IMAU), Menyuan County, Zhugu Town, Sigou, 2616 m, 37°07'56.19"N, 102°24'03.91"E, 24.VII.2021, leg. Li Shi; • 8♂♂12♀♀ (IMAU), Qilian County, Zhamashi Town, Xigou, 3196 m, 38°08'32.91"N, 99°58'12.59"E, 03.VIII.2021, leg. Li Shi; • 6♂♂14♀♀ (IMAU), Qilian County, Yeniugou Town, Yanglong Town, near Xiage’er Snow Mountains, 3313.3 m, 38°48'30.32"N, 98°23'48.35"E, 04.VIII.2021, leg. Li Shi; • 5♂♂17♀♀ (IMAU), Qilian County, Babao Town, Binggou Village, Xiaxigou, 3170 m, 38°07'50.17"N, 100°09'57.18"E, 05.VIII.2021, leg. Li Shi; • 3♂♂1♀♀ (IMAU), Qilian County, Zhamashi Town, Donggou, 3106 m, 38°09'10.99"N, 100°01'26.85"E, 03.VIII.2021, leg. Li Shi; • 27♂♂27♀♀ (IMAU), Qilian County, Lujiaogou, 3417 m, 38°06'31.22"N, 100°28'42.33"E, 01.VIII.2021, leg. Li Shi. DNA sequence number PX103170 from GenBank.

#### Diagnosis.

Frons with three *ori* inclinate and two *ors* reclinate surrounded by faint brown at base. Antenna with yellow scape and pedicel, first flagellomere mostly brown with anterodistal corner like a short spine. Mesonotum mostly black except for rectangular part yellow above notopleuron, postpronotum black on anterior half and yellow on posterior half; 1+2 *dc.* Legs mostly black except for fore coxae yellow at apex and all femora yellow on apical 5/6. Distiphallus gourd-shaped with enlarged apex and complete ventral opening.

#### Redescription.

**Figure 2.**
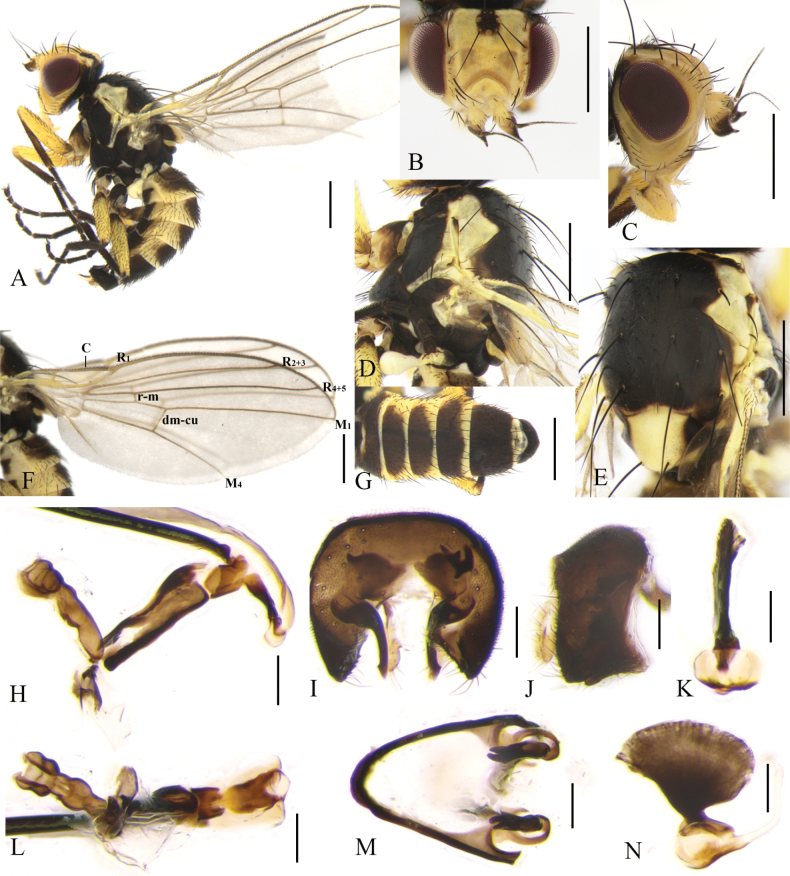
Cerodontha (Cerodontha) flavicornis (Egger, 1862). Male. **A.** Habitus, lateral view; **B, C.** Head, dorsal and lateral view; **D, E.** Thorax, lateral and dorsal view; **F.** Wing; **G.** Abdomen, dorsal view; **H, L.** Phallic complex, lateral and ventral view; **I, J.** Epandrial complex, anterior and lateral view; **K, N.** Ejaculatory apodeme, ventral and lateral view; **M.** Hypandrium, ventral view. Scale bars: 0.5 mm (**A–E**); 0.1 mm (**H–N**).

**Figure 3.**
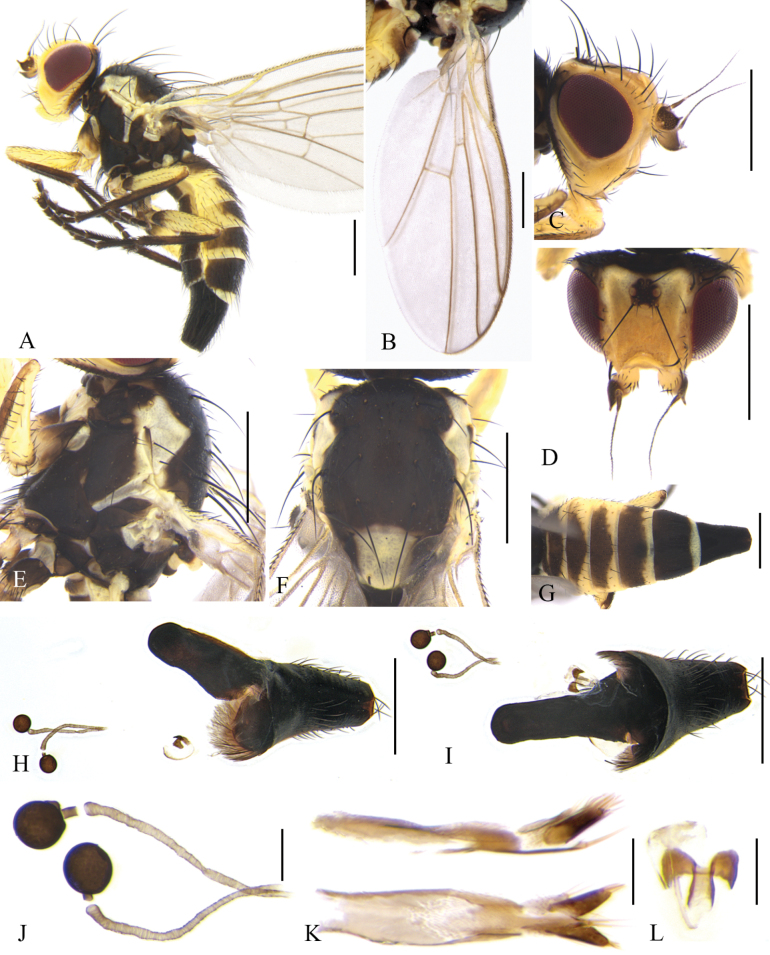
Cerodontha (Cerodontha) flavicornis (Egger, 1862). Female. **A.** Habitus, lateral view; **B.** Wing; **C, D.** Head, lateral and dorsal view; **E, F.** Thorax, lateral and dorsal view; **G.** Abdomen, dorsal view; **H, I.** Spermathecae, ventral receptacle and oviscape, lateral and ventral view; **J.** Spermathecae; **K.** Proctiger, lateral and ventral view; **L.** Ventral receptacle, ventral view. Scale bars: 0.5 mm (**A–I**); 0.1 mm (**J–L**).

**Male** (Fig. 2A–N). Body length 2.3–3.0 mm; wing length 2.7–3.2 mm. Female (Fig. 3A–L). Body length 2.5–3.6 mm; wing length 2.8–3.4 mm.

Head (Fig. 2B, C) light yellow. Frons wider than high, conspicuously projecting above eye in lateral view, ~1.1 × width of eye in dorsal view; three *ori* inclinate and two *ors* reclinate surrounded by faint brown coloration at base; orbital setulae slender in one inclinate row near *ori*; inner vertical setae surrounded by yellowish brown coloration at base; outer vertical setae on black background. Ocellar triangle dark brown, ocellar setae longer distinctly than *ors.* Lunule 2.3 × wider than high and upper margin brown near level of posterior *ori.* Antenna mostly yellow, first flagellomere mostly brown with anterodistal corner like a short spine, arista brown with microscopic pubescence. Gena ~2/5 height of eye. Clypeus and palpus light yellow.

Thorax (Fig. 2D, E) mostly black. Mesonotum mostly black except for rectangular part yellow above notopleuron, postpronotum black on anterior half and yellow on posterior half; 1+2 *dc*; *acr* in 2–4 irregular rows, one postsutural intra-alar seta, one presutural and two postsutural supra-alar setae. Notopleuron yellow. Anepisternum black except dorsal and posterior margin yellow, with one strong anepisternal seta, four long setulae and two short setulae. Katepisternum black with one strong katepisternal seta and six short setulae. Scutellum yellow but dark brown at lateral corner. Legs mostly black, fore coxae yellow at apex and all femora yellow on apical 5/6. Wing: Costa with 2^nd^ (between R_1_ and R_2+3_), 3^rd^ (between R_2+3_ and R_4+5_) and 4^th^ (between R_4+5_ and M_1_) sections in proportion of 4.4:1.3:1; ultimate and penultimate sections of M_1_ in proportion of 2.3:1; ultimate and penultimate sections of M_4_ in proportion of 1.3:1; *r-m* at apical 1/3 of discal cell. Calypter yellow with margin and fringe dark brown. Halter pale yellow.

Abdomen (Fig. 2G) mostly yellow with wide black stripes in the middle, tergite 6 entirely black. Male genitalia (Fig. 2H–N): epandrium rounded with strong dorsal and lateral setae; surstylus with sparse and short spines; mesophallus twice length of distiphallus, with ventral opening at distal 1/4; distiphallus gourd-shaped with enlarged apex and complete ventral opening; ejaculatory apodeme asymmetrical, blade clear and broad; sperm pump with conspicuously brown bottom connected with base of duct. Cercus lightly sclerotized.

**Female.** Tergite 6–7 entirely black. Other external characteristics (Fig. 3A–G) same as the male except for the female terminalia. Terminalia (Fig. 3H–L): two spermathecae equal in size, circular and truncated at basal 1/6; duct long, with helical texture and a short hyaline segment near base of spermathecae; ventral receptacle (Fig. 3L) circular in lateral view, and butterfly-shaped in ventral view; proctiger constricted on apical 1/3 (Fig. 3K). Cercus setose, relatively long and well-sclerotized.

#### Distribution.

Palaearctic: Albania, Andorra, Austria, Belgium, China (Qinghai)^#^, Croatia (Černý 2018), France, Germany, Hungary, Italy, Montenegro (Spasić 1996), Poland, Russia, Spain, Switzerland (Černý and Bächli 2018).

#### Remarks.

Among the Palaearctic species of subgenus Cerodontha, this species is distinctly characterized by its antennal shape and coloration, the black mesonotum, the stripes on the abdominal tergites, and the distinctive shape of the distiphallus. The species is similar to C. (C.) affinis (Fallén, 1823) from the Palaearctic region in the following characters: mesonotum black; notopleuron, apical half of postpronotum, dorsal margin of anepisternum and the middle of scutellum yellow; *acr* in two rows; calypter yellow with margin and fringe dark brown; femora of yellow. But it can be differentiated from the latter by the first flagellomere being yellowish with a spine at upper corner; mesonotum having 1+2 *dc*; legs having black tibiae and tarsi; abdomen being mostly yellow with wide black stripes in the middle, and the tergite 6 being entirely black. In C. (C.) affinis, the first flagellomere is black with blunt projection at dorsal corner (Spencer 1976: fig. 316); the mesonotum has 1+3 *dc*; the fore tibiae are yellowish brown; the abdomen is shining black but all tergites yellow-bordered and also laterally yellow in male.

### ﻿ Cerodontha (Cerodontha) fulvipes

Taxon classificationAnimaliaDipteraAgromyzidae

(Meigen, 1830)

DBD838AC-66E6-5BA6-9203-55722A6A3F0F

Figs 4A–M, 5A–L

Agromyza fulvipes Meigen, 1830: 174.
Agromyza femoralis Meigen, 1838: 397.
Agromyza occulta Meigen, 1838: 403; Schiner 1964: 307.
Odontocera spinicornis Macquart, 1835: 615; Hendel 1920: 170.
Ceratomyza fulvipes : Becker 1902: 339.
Cerodonta fulvipes : Hendel 1920: 169.
Cerodontha (Cerodontha) fulvipes : Sasakawa 1961: 388; Nowakowski 1967: 657; 1972: 739; 1973: 58; Griffiths 1968: 78; Rohdendorf 1970: 270; Spencer 1972: 107; Spencer 1976: 179; Soós and Papp 1984: 286; Spasić 1996: 114; Černý et al. 2018: 21; Černý et al. 2022.

#### Specimens examined.

China, Qinghai Province: • 1♀ (IMAU), Haidong City, Huzhu County, Bazha Town, Baimuxia Village, 3070 m, 37°00'09.38"N, 102°07'36.04"E, 24.VII.2021, leg. Li Shi; • 2♂♂ (IMAU), Menyuan County, Zhugu Town, Sigou, 3344 m, 37°06'16.46"N, 102°36'04.03"E, 28.VII.2021, leg. Li Shi; • 1♂11♀♀ (IMAU), Menyuan County, Xianmi Town, Qiaotan Village, Qihankaigou, 2715 m, 37°09'33.51"N, 102°02'05.13"E, 30.VII.2021, leg. Li Shi; • 1♂ (IMAU), Menyuan County, Zhugu Town, Dezong Village, 2894 m, 37°13'02.69"N, 102°11'58.20"E, 26.VII.2021, leg. Li Shi; • 1♂1♀ (IMAU), Qilian County, Zhamashi Town, Donggou, 3106 m, 38°09'10.99"N, 100°01'26.85"E, 03.VIII.2021, leg. Li Shi; • 1♀ (IMAU), Qilian County, Zhamashi Town, Xigou, 3196 m, 38°08'32.91"N, 99°58'12.59"E, 03.VIII.2021, leg. Li Shi; • 1♀ (IMAU), Qilian County, Lujiaogou, 3417 m, 38°06'31.22"N, 100°28'42.33"E, 01.VIII.2021, leg. Li Shi; • 1♂ (IMAU), Qilian County, Babao Town, Binggou Village, Xiaxigou, 3170 m, 38°07'50.17"N, 100°09'57.18"E, 05.VIII.2021, leg. Li Shi. DNA sequence number PX102191 from GenBank.

#### Diagnosis.

**Figure 4.**
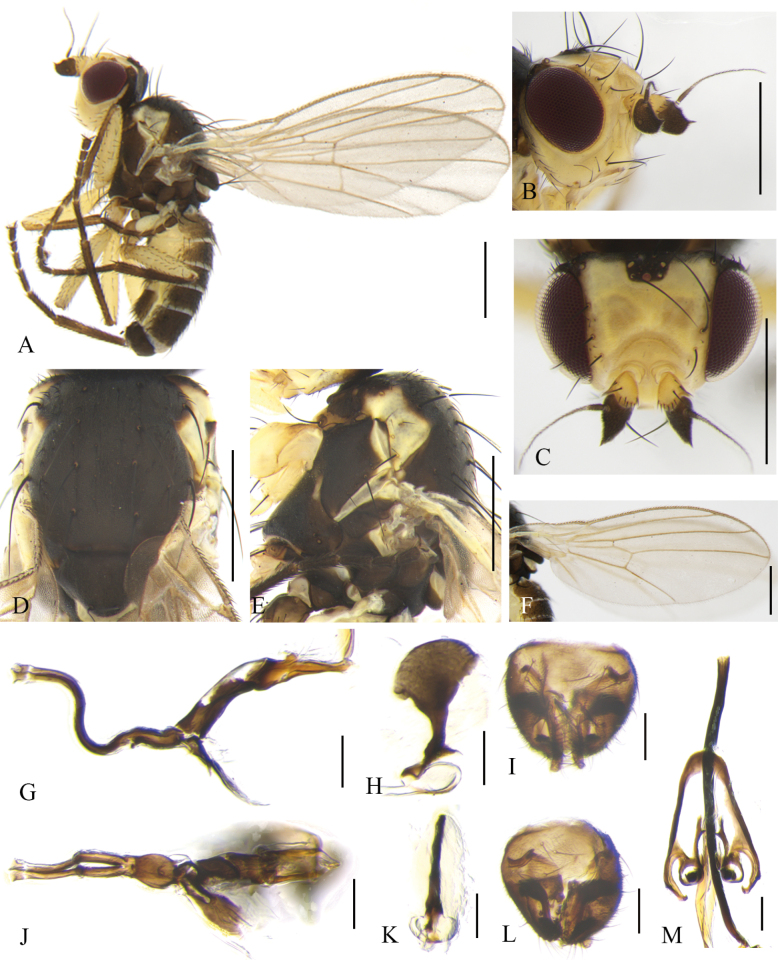
Cerodontha (Cerodontha) fulvipes (Meigen, 1830). Male. **A.** Habitus, lateral view; **B, C.** Head, lateral and dorsal view; **D, E.** Thorax, dorsal and lateral view; **F.** Wing. **G, J.** Phallic complex, lateral and ventral view; **H, K.** Ejaculatory apodeme, lateral and ventral view; **I, L.** Epandrial complex, anterior and anterolateral view; **M.** Hypandrium, ventral view. Scale bars: 0.5 mm (**A–F**); 0.1 mm (**G–M**).

**Figure 5.**
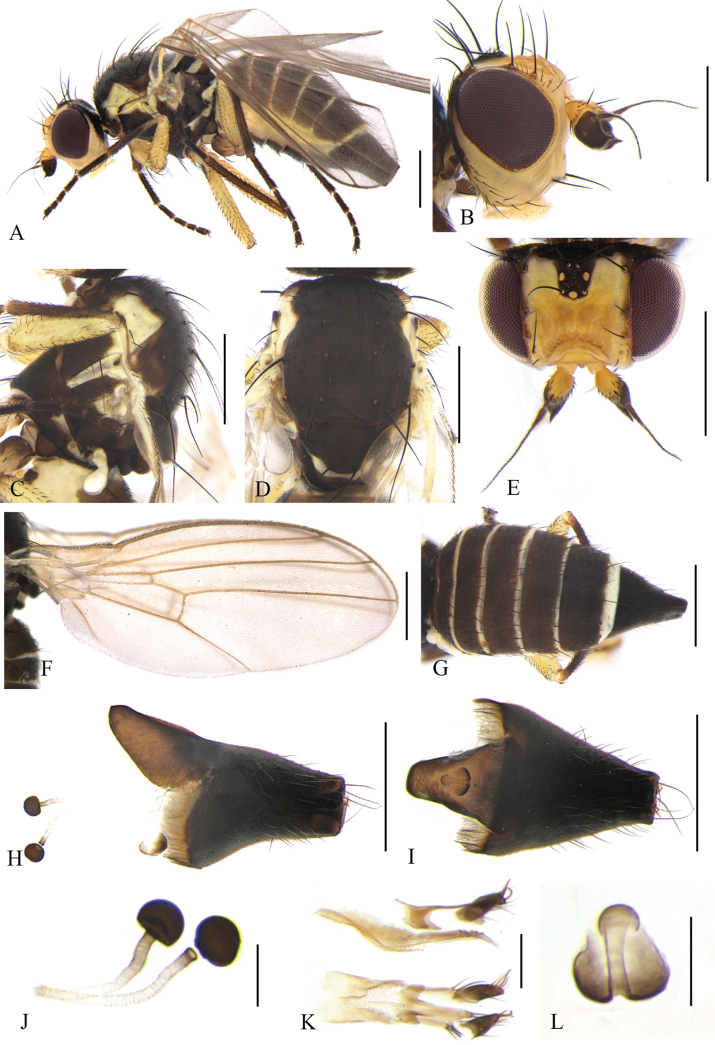
Cerodontha (Cerodontha) fulvipes (Meigen, 1830). Female. **A.** Habitus, lateral view; **B, E.** Head, lateral and dorsal view; **C, D.** Thorax, lateral and dorsal view; **F.** Wing; **G.** Abdomen, dorsal view; **H, I.** Spermathecae, ventral receptacle and oviscape, lateral and ventral view; **J.** Spermathecae; **K.** Proctiger, lateral and ventral view; **L.** Ventral receptacle, ventral view. Scale bars: 0.5 mm (**A–I**); 0.1 mm (**J–L**).

Male (Fig. 4A–M). Body length 1.9–2.4 mm; wing length 1.9–2.5 mm. Female (Fig. 5A–L). Body length 2.1–2.9 mm; wing length 2.2–2.8 mm. Fronto-orbital plate light brown or dark brown between two *ors.* Inner vertical setae surrounded by yellow coloration at base. First flagellomere brownish black, distinctly angulate at anterodistal corner; arista thickened on basal 1/5. Gena ~2/3 height of eye; genal grooves distinct on anterior 1/2 of gena. Mesonotum and scutellum shiny black, *acr* in two irregular rows (Fig. 4D), supra-alar setae on dark brown background. Anepisternum black except for yellow dorsal and posterior margin. Katepisternum black. Legs yellow with tibiae and tarsi brown. Male genitalia (Fig. 4G–M): distiphallus S-shaped with distal tubules parallel, ~2.5 × length of mesophallus; terminal processes short, pale brown. Female terminalia (Fig. 5H–L): spermathecae circular and truncated at basal 1/4, internal duct invagination 1/4 as deep as height of spermathecae.

#### Distribution.

Palaearctic: Austria, Belgium, Bulgaria (Černý et al. 2022), China (Xinjiang, Qinghai*), Croatia, Czech Republic, Denmark, Estonia, Finland, France, Germany, Greece, Hungary, Ireland, Italy, Japan, Latvia, Lithuania, Montenegro (Spasić 1996), Netherlands, Norway, Poland, Portugal (Černý et al. 2018), Romania, Russia, Slovakia, Spain, Sweden, Tajikistan, The United Kingdom, Turkey, Ukraine, Uzbekistan (Soós and Papp 1984).

#### Remarks.

The new species is similar to C. (C.) denticornis (Panzer, 1806) from the Palaearctic, Oriental and Afrotropical regions in the first flagellomere brownish black with a spine at dorsal corner, but it can be separated from the latter by mesonotum and scutellum being shiny black; *acr* being in two irregular rows; the distiphallus (Fig. 4G, J) having trumpet-shaped terminal processes less pronounced than C. (C.) denticornis (Spencer 1976: fig. 319). In C. (C.) denticornis, the mesonotum is matt grayish black or yellow centrally and adjoining the scutellum; the scutellum is either black or variably yellow; the *acr* rows are absent.

### ﻿Subgenus Dizygomyza Hendel, 1920

#### ﻿ Cerodontha (Dizygomyza) flavilunulata sp. nov.

Taxon classificationAnimaliaDipteraAgromyzidae

E03DA870-A122-5284-B6B2-F7F0093B9A6D

https://zoobank.org/E576FD2B-1E8D-4F3C-861B-6E2B19ABCF91

Figs 6A–O, 7A–K

##### Type material.

China, Inner Mongolia, Genhe City, Mangui Town, the northern primitive forest region of Greater Khingan Mountains: ***Holotype*.** • ♂ (IMAU), Wulonggan forestry center, near impounding reservoir, unburned area, Pinus sylvestris var. mongholica, malaise trap 20 (one meter above the ground), 52°47'47.98"N, 120°55'03.45"E, 816 m, 13.VII.2022, leg. Li Shi, Zhi-Wei Wang, Rui Ma. ***Paratypes*.** • 5♂♂ (IMAU), same data as for holotype; • 1♀ (IMAU), Wulonggan forestry center at 4217 meters, unburned area, *Larix gmelinii*, malaise trap-14 (one meter above the ground), 52°47'05.26"N, 120°53'40.10"E, 789 m, 28.VII.2022, leg. Rui Ma, Qin-Jianrong Liu; • 1♂ (IMAU), Wulonggan forestry center near impounding reservoir, unburned area, Pinus sylvestris var. mongholica, malaise trap 19 (three meters above the ground), 52°47'47.98"N, 120°55'03.45"E, 816 m, 28.VII.2022, leg. Rui Ma, Qin-Jianrong Liu; • 1♂ (IMAU), Aba River Third Branch Line, unburned area, Pinus sylvestris var. mongholica, malaise trap 34 (one meter above the ground), 52°22'00.00"N, 120°27'07.00"E, 659 m, 28.VII.2022, leg. Rui Ma, Qin-Jianrong Liu; • 1♂ (IMAU), Changliangbeishan near stock ground, unburned area, *Pinus pumila*, malaise trap 10 (one meter above the ground), 52°28'55.60"N, 121°02'40.50"E, 937 m, 28.VII.2022, leg. Rui Ma, Qin-Jianrong Liu; • 2♂♂ (IMAU), Changliangbeishan houdu, burned area in 2002, Pinus sylvestris var. mongholica, malaise trap 6 (one meter above the ground), 52°26'01.00"N, 120°56'53.20"E, 985 m, 14.VII.2022, leg. Li Shi, Zhi-Wei Wang, Rui Ma. DNA sequence number PX103169 from GenBank.

##### Diagnosis.

Frons mostly yellow with variable black stripes and spots at levels of between posterior *ori* and posterior *ors.* Lunule mostly yellow with brown upper margin, sometimes mostly brown with tiny yellow spots, wider than high. Mesonotum with gray pruinosity and a pair of prescutellar setae. Legs brown, all femora yellow on apical 1/6, all knees yellow, and all tibiae yellow on basal 1/7. Calypter and margin yellow, fringe yellowish white. Abdomen brown, tergites 1–6 yellowish on posterior and lateral margin. Distiphallus with subparallel S-shaped tubules.

##### Description.

**Figure 6.**
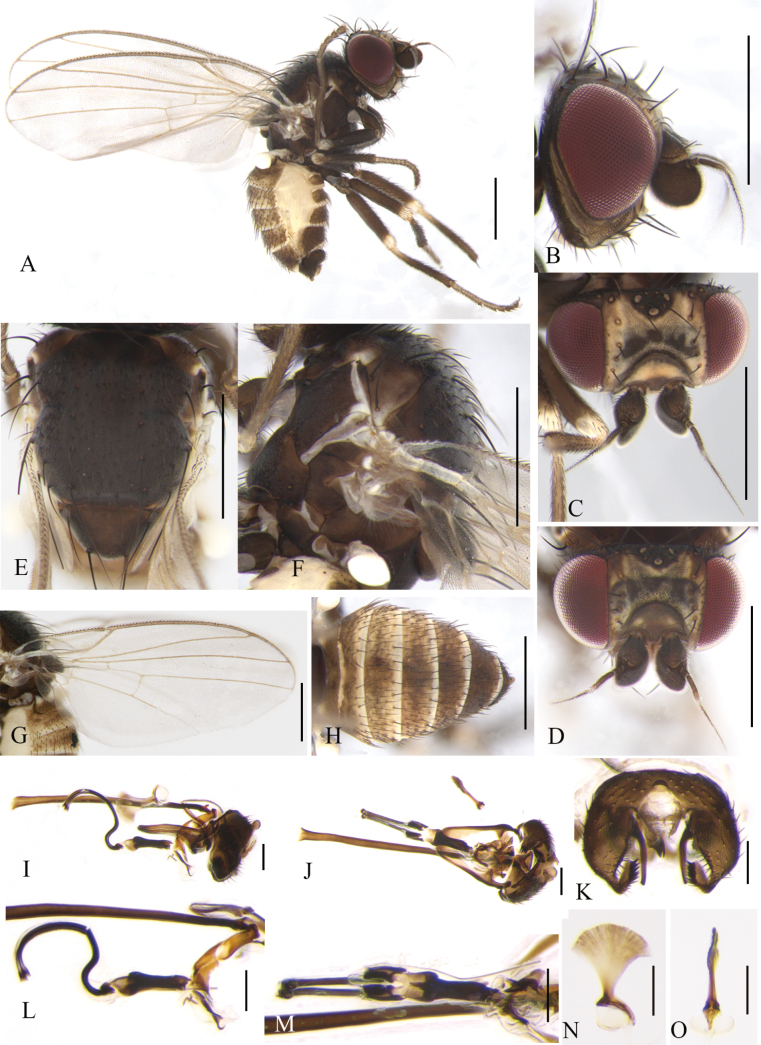
Cerodontha (Dizygomyza) flavilunulata sp. nov. Paratype male. **A.** Habitus, lateral view; **B, C, D.** Head, lateral and dorsal view; **E, F.** Thorax, dorsal and lateral view; **G.** Wing; **H.** Abdomen, dorsal view; **I, J.** Genitalia, lateral and ventral view; **K.** Epandrial complex, dorsal view; **L, M.** Phallic complex, lateral and ventral view (S-shaped tubules of distiphallus with a minor crack); **N, O.** Ejaculatory apodeme, lateral and ventral view. Scale bars: 0.5 mm (**A–H**); 0.1 mm (**I–O**).

**Figure 7.**
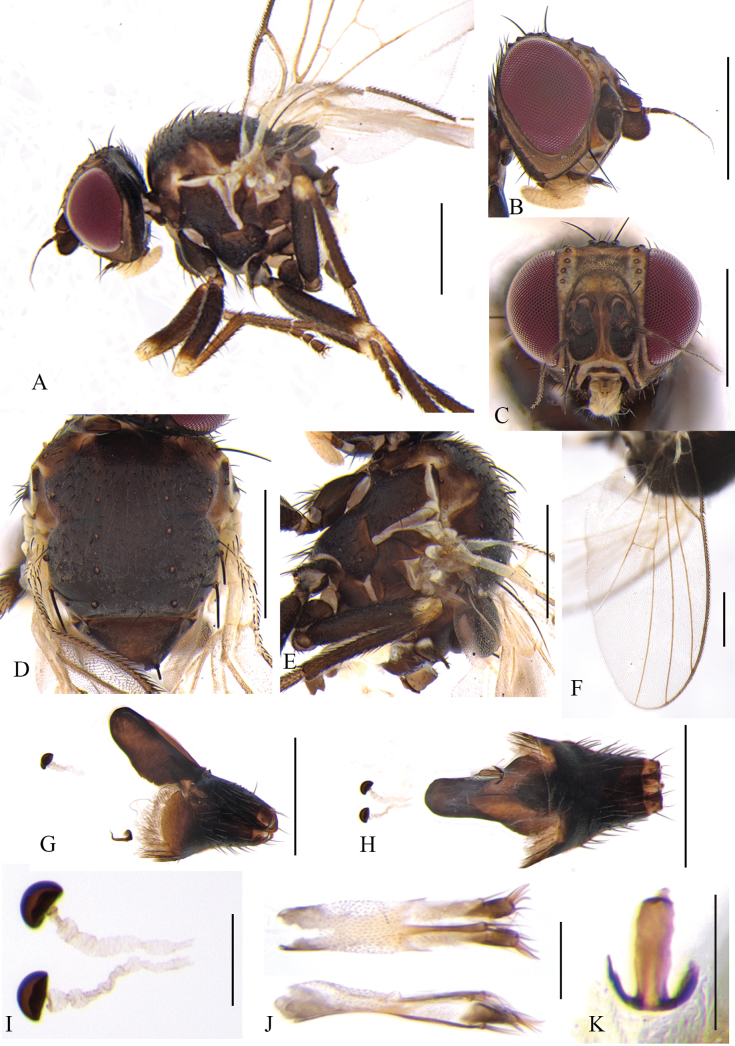
Cerodontha (Dizygomyza) flavilunulata sp. nov. Paratype female. **A.** Habitus, lateral view. **B, C.** Head, lateral and dorsal view; **D, E.** Thorax, dorsal and lateral view; **F.** Wing; **G, H.** Spermathecae, ventral receptacle and oviscape, lateral and ventral view; **I.** Spermathecae; **J.** Proctiger, lateral and ventral view; **K.** Ventral receptacle, ventral view. Scale bars: 0.5 mm (**A–H**); 0.1 mm (**I–K**).

**Male** (Fig. 6A–O). Body length 1.9–2.1 mm; wing length 2.1–2.5 mm. Female (Fig. 7A–K). Body length 2.1 mm; wing length 2.3 mm.

Head (Fig. 6B–D) mostly yellow. Frons with grayish pruinosity, variable black stripes and spots at levels of between posterior *ori* and posterior *ors*, and frons projecting above eye in lateral view, ~0.9 × as wide as eye in dorsal view; fronto-orbital plate dark yellow and slightly shiny, becoming brown beyond posterior *ors*, and ~1/3 width of frons; two inclinate *ori* and two reclinate *ors* surrounded by brown coloration; orbital setulae sparse, reclinate in a single row. Ocellar triangle black, ocellar setae distinctly longer than posterior *ors.* Lunule mostly yellow with brown upper margin (Fig. 6C), sometimes mostly brown with tiny yellow spots (Fig. 6D), wider than high. Antenna brownish black, first flagellomere enlarged with brownish pubescence; arista conspicuously thickened on basal 1/4–1/3. Face brown, facial keel broad between antennae. Gena narrow, dark yellow, ~1/5 eye height. Clypeus pale yellow, palpus brown.

Thorax (Fig. 6E, F) black. Mesonotum with gray pruinosity and moderately shiny, but yellow at posterolateral corner of postpronotum; 1+3 *dc*, *acr* in six irregular rows, a pair of prescutellar setae, two postsutural intra-alar, one presutural and two strong postsutural supra-alar setae on dark brown background. Notopleuron brown except for pale yellow at posteroventral corner. Anepisternum dark brown except for yellow dorsal margin, with one strong anepisternal seta and 7–9 short setulae. Katepisternum black with one strong katepisternal seta and four short setulae. Legs brown, all femora yellow on apical 1/6, all knees yellow, all tibiae yellow on basal 1/7, and all tarsi yellowish brown. Wing: Costa with 2^nd^ (between R_1_ and R_2+3_), 3^rd^ (between R_2+3_ and R_4+5_), and 4^th^ (between R_4+5_ and M_1_) sections in proportion of 5:1.6:1; ultimate and penultimate sections of M_1_ in proportion of 2.2:1; *r-m* slightly beyond middle of discal cell; ultimate and penultimate sections of M_4_ in proportion of 1.6–1.1:1. Calypter and margin yellow, fringe yellowish white. Halter yellowish white.

Abdomen (Fig. 6A, H) brown, tergites 1–6 yellowish on posterior and lateral margin. Genitalia (Fig. 6I–O): epandrium with a pair of long claviform processes in posterior view; surstylus with nine long spines in inner margin; mesophallus paler, dilated at apex, with ventral edge curved obviously; distiphallus with long subparallel S-shaped tubules, basal curve deep and not recurved apically; ejaculatory apodeme symmetrical, blade brownish, stem dark; sperm pump pale but base of duct lightly pigmented.

**Female** (Fig. 7A–K). Lunule narrower than that of the male. First flagellomere not as large as that of male (Fig. 7B, C). Abdomen brown, tergites 1–7 yellowish on posterior and lateral margin. Other external characteristics same as the male except for the female terminalia. Terminalia (Fig. 7G–K): spermathecae (Fig. 7I) blackish brown, mushroom-shaped; spermathecal duct wide, weakly sclerotized with wavy lines; ventral receptacle symmetrical with wide stem; proctiger constricted medially in lateral view. Cercus well-sclerotized.

##### Distribution.

China (Inner Mongolia).

##### Etymology.

The specific name compounds the Latin prefix *flavi*- (meaning yellow) and the Latin noun *lunulata* (meaning lunule), referring to the mostly yellow lunule.

##### Remarks.

The new species is similar to C. (D.) morosa from the Palaearctic, Nearctic, and Oriental regions in the following characteristics: wing with ultimate section of M_4_ slightly longer than penultimate section; calypter margin and fringe pale yellow or white; legs with all knees yellow; epandrium with a pair of long claviform processes in posterior view. But it can be distinguished by the following characteristics: the frons being yellow having grayish pruinosity, variable black stripes and spots at levels of between posterior *ori* and posterior *ors*; the fronto-orbital plate being dark yellow and slightly shiny, becoming brown beyond posterior *ors*; the lunule being mostly yellow with brown upper margin, sometimes mostly brown with tiny yellow spots; the mesonotum having a pair of prescutellar setae; the tubules of the distiphallus having deep basal curve and not recurved apically. In C. (D.) morosa, the frons is dark brown to black; the fronto-orbital plate is dark brown, but sometimes brown with darker pigment reduced to the posterolateral vestige; the lunule is smooth and velvety grayish; the mesonotum has *acr* in four rows; the prescutellar setae are absent; the distiphallus has shallow basal curve but more recurved apically (Spencer 1976; Lonsdale 2021).

#### ﻿ Cerodontha (Dizygomyza) granditerga sp. nov.

Taxon classificationAnimaliaDipteraAgromyzidae

825533E2-A678-503F-BB51-1CA8B2AA034E

https://zoobank.org/EA6A1251-F9B7-4E8B-971F-3DE0D498AA43

Fig. 8A–M

##### Type material.

China, Qinghai Province: ***Holotype*.** • ♂ (IMAU), Menyuan County, Zhugu Town, Sigou, 3344 m, 37°06'16.46"N, 102°36'04.03"E, 28.VII.2021, leg. Li Shi. ***Paratypes*.** • 1♂ (IMAU), same data as for holotype; • 2♂♂ (IMAU), Qilian County, Lujiaogou, 3417 m, 38°06'31.22"N, 100°28'42.33"E, 01.VIII.2021, leg. Li Shi. DNA sequence number PX103174 from GenBank.

##### Diagnosis.

Fronto-orbital plate blackish brown, moderately shiny, ~1/3 width of frons; two inclinate *ori* and two reclinate *ors.* Lunule brown, twice as wide as high, projecting above frons in lateral view. Calypter and margin yellow, fringe brown. Epandrium broad with a long downward-curved spine on inner margin of dorsal 3/4; surstylus directed inwards, with ~25 short and thick spines; distiphallus short S-shaped in lateral view, and divided into one pair of unparallel tubules, approx. as long as mesophallus in ventral view.

##### Description.

**Figure 8.**
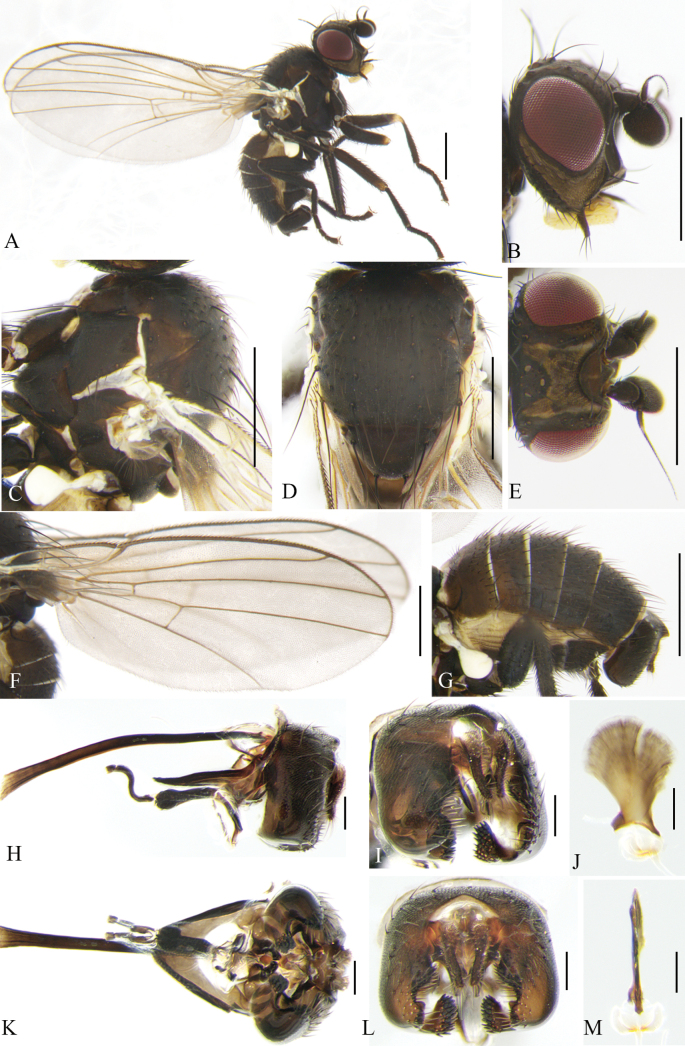
Cerodontha (Dizygomyza) granditerga sp. nov. Paratype male. **A.** Habitus, lateral view; **B, E.** Head, lateral and dorsal view; **C, D.** Thorax, dorsal and lateral view; **F.** Wing; **G.** Abdomen, lateral view; **H, K.** Genitalia, lateral and ventral view; **I, L.** Epandrial complex, posterolateral and posterior view; **J, M.** Ejaculatory apodeme, lateral and ventral view. Scale bars: 0.5 mm (**A–G**); 0.1 mm (**H–M**).

**Male** (Fig. 8A–M). Body length 2.0–2.3 mm; wing length 2.5–2.8 mm.

Head (Fig. 8B, E) blackish brown. Frons dark brown, projecting slightly above eye in lateral view, and ~1.7 × as wide as eye in dorsal view; fronto-orbital plate blackish brown, moderately shiny, ~1/3 width of frons; two inclinate *ori* and two reclinate *ors*; orbital setulae sparse and thin, reclinate in a single row. Ocellar triangle black; ocellar setae conspicuously weaker than *ors.* Lunule brown, twice as wide as high, projecting above frons in lateral view. Antennal first flagellomere enlarged with pale pubescence; arista conspicuously thickened on basal 1/4. Facial keel distinctly raised. Gena yellowish brown, ~1/2 height of eye, highest point located at middle of eye. Clypeus pale yellow; palpus black with three prominent setae at apex.

Thorax (Fig. 8C, D) black. Mesonotum and scutellum moderately shiny; 1+3 *dc*; *acr* in four irregular rows; two postsutural intra-alar, one presutural and two strong postsutural supra-alar setae. Anepisternum with one anepisternal seta, two long setulae and six short setulae. Katepisternum with one katepisternal seta and five short setulae. Legs black, only fore femora with yellow knees. Wing: Costa with 2^nd^ (between R_1_ and R_2+3_), 3^rd^ (between R_2+3_ and R_4+5_) and 4^th^ (between R_4+5_ and M_1_) sections in proportion of 4.7:1.9:1; ultimate and penultimate sections of M_1_ in proportion of 2.2:1; *r-m* slightly beyond middle of discal cell; ultimate and penultimate sections of M_4_ in proportion of 1:1. Calypter and margin yellow, fringe brown. Halter pale yellow.

Abdomen (Fig. 8G) dark brown, tergites 1–6 yellowish on posterior margin. Genitalia (Fig. 8H–M): epandrium broad with a long downward-curved spine on inner margin of dorsal 3/4; surstylus with ~25 short and thick spines; mesophallus dark completely, broad on apical 1/3; mesophallus and distiphallus wrapped in a layer of pale membrane; distiphallus short S-shaped in lateral view, and divided into one pair of unparallel tubules, approx. as long as mesophallus in ventral view; ejaculatory apodeme with blade brown and stem wide; sperm pump yellowish white. Cercus with a long strong seta downward.

**Female.** Unknown.

##### Distribution.

China (Qinghai).

##### Etymology.

The specific name of this new species compounds the Latin prefix *grandi*- (meaning large) and the Latin noun *terga* (meaning tergite), referring to the notably broad epandrium.

##### Remarks.

The new species has the relatively largest epandrial complex which can be easily distinguished from other species of the subgenus Dizygomyza.

#### ﻿ Cerodontha (Dizygomyza) labradorensis

Taxon classificationAnimaliaDipteraAgromyzidae

Spencer, 1969

21ED4F9E-56EE-5C7A-BCE4-7D07ABAD0AE4

Fig. 9A–M

Cerodontha (Dizygomyza) labradorensis Spencer, 1969: 120.
Cerodontha (Dizygomyza) poolei Spencer & Steyskal, 1986: 284.
Cerodontha (Dizygomyza) orbitalis Zlobin, 1984b: 520; 1996: 281.

##### Specimens examined.

China, Qinghai Province: • 1♂ (IMAU), Menyuan County, Zhugu Town, Sigou, 3344 m, 37°06'16.46"N, 102°36'04.03"E, 28.VII.2021, leg. Li Shi; • 1♂ (IMAU), Qilian County, Lujiaogou, 3417 m, 38°06'31.22"N, 100°28'42.33"E, 01.VIII.2021, leg. Li Shi.

##### Diagnosis.

Frons and parafacial projecting above eye. Fronto-orbital plate brownish black, moderately shiny, inner margin yellow, ~2/3 width of frons; three *ori* and two *ors.* Lunule brown with distinctly shining, slightly sunken, broadly semicircular, approx. wider than high. Antennal arista conspicuously thickened on basal 1/2. Leg black, only fore femora with yellow knees. Wing with ultimate and penultimate sections of M_4_ in proportion of 1.1:1. Calypter and fringe yellow, margin brownish yellow. Surstylus directed inwards, with 5 thick spines on inner margin; distiphallus S-shaped, ~2.5 × length of mesophallus and yellowish brown at apex in lateral view.

##### Redescription.

**Figure 9.**
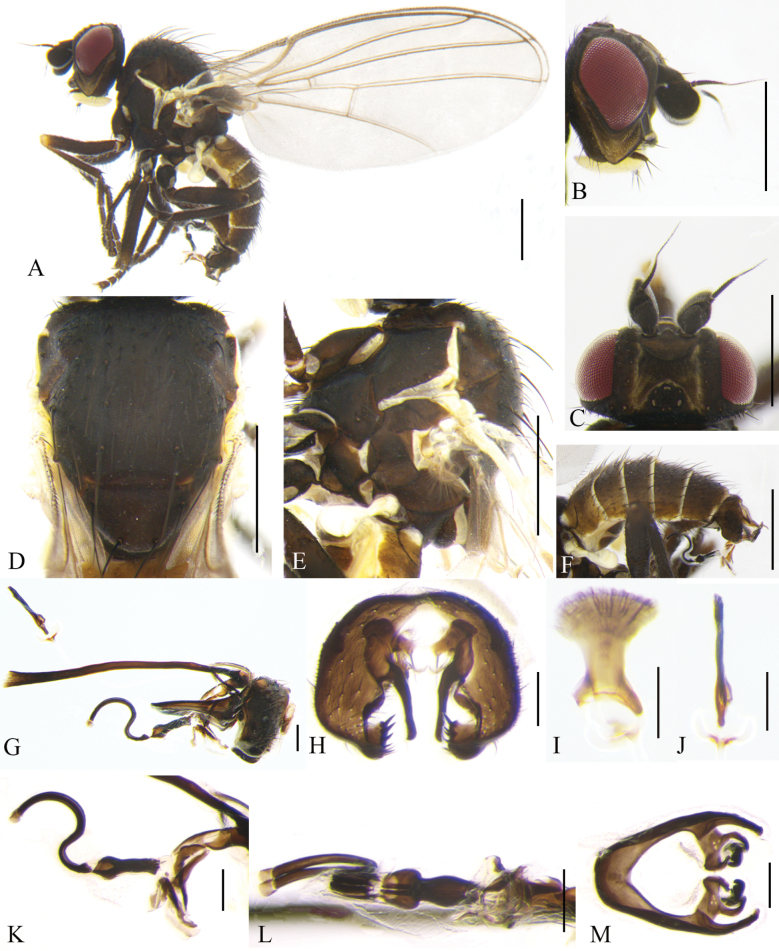
Cerodontha (Dizygomyza) labradorensis Spencer, 1969. Male. **A.** Habitus, lateral view; **B, C.** Head, lateral and dorsal view; **D, E.** Thorax, dorsal and lateral view; **F.** Abdomen, lateral view; **G.** Genitalia, lateral view; **H.** Epandrial complex, anterior view; **I, J.** Ejaculatory apodeme, lateral and ventral view; **K, L.** Phallic complex, lateral and ventral view; **M.** Hypandrium, ventral view. Scale bars: 0.5 mm (**A–F**); 0.1 mm (**G–M**).

**Male.** Body length 2.3–2.7 mm; wing length 2.4–2.7 mm (Fig. 9A).

Head (Fig. 9B, C) dark brown. Frons and parafacial projecting above eye; frons dark brown, ~1.4 × as wide as eye; fronto-orbital plate brownish black, moderately shiny, inner margin yellow, ~2/3 width of frons; three *ori* and two *ors*; orbital setulae reclinate or erect in a single row. Ocellar triangle black with yellow margin; ocellar setae conspicuously weaker than *ors.* Lunule brown with distinctly shining, slightly sunken, broadly semicircular, approx. wider than high. Antennal first flagellomere enlarged with pale pubescence; arista conspicuously thickened on basal 1/2. Facial keel distinctly raised. Gena brownish yellow in the center and dark brown on both sides, ~2/5 height of eye, highest point located at middle of eye. Clypeus light yellow; palpus black, three prominent setae at apex.

Thorax (Fig. 9D, E) black. Mesonotum and scutellum black, moderately shiny; 1+3 *dc*; *acr* in four irregular rows; one postsutural intra-alar, one presutural and two strong postsutural supra-alar setae. Anepisternum with one anepisternal seta, eleven short setulae. Katepisternum with one katepisternal seta and four short setulae. Leg black, only fore femora with yellow knees. Wing: Costa with 2^nd^ (between R_1_ and R_2+3_), 3^rd^ (between R_2+3_ and R_4+5_) and 4^th^ (between R_4+5_ and M_1_) sections in proportion of 4.1:1.4:1; ultimate and penultimate sections of M_1_ in proportion of 1.9:1; *r-m* slightly beyond middle of discal cell; ultimate and penultimate sections of M_4_ in proportion of 1.1:1. Calypter and fringe yellow, margin brownish yellow. Halter pale yellow.

Abdomen (Fig. 9F) black, tergites 2–6 with yellow posterior margin. Genitalia (Fig. 9G–M): epandrium (Fig. 9H) setose, with a pair of distinctly long claviform processes; surstylus directed inwards, with five thick spines on inner margin; mesophallus swollen at distal 1/3 with a medial suture in ventral view; distiphallus S-shaped, ~2.5 × length of mesophallus, and yellowish brown at apex in lateral view; ejaculatory apodeme with conspicuously wide stem, blade margin clear; sperm pump pale but base of duct lightly pigmented. Cercus with a long seta in posterior view.

**Female.** Unknown.

##### Distribution.

Palaearctic: China (Qinghai)^#^, Kirgizia, Mongolia (Zlobin 1996), Russia, Tajikistan, Uzbekistan; Nearctic: USA, Canada.

##### Remarks.

The species is similar to C. (D.) luctuosa in the following characteristics: frons dark brown; arista conspicuously thickened on basal 1/2; wing with ultimate section of M_4_ equal or slightly longer than penultimate. Legs black, only fore knees yellow; abdomen black and tergites 2–6 with yellow posterior margin; epandrium with a pair of distinctly long claviform processes; distiphallus S-shaped; mesophallus swollen apically. However, it can be distinguished by the following characteristics: the calypter and fringe being yellow, margin brownish yellow; the distiphallus being ~2.5 × length of mesophallus, and yellowish brown at apex in lateral view. In C. (D.) luctuosa, the calypter and fringe are whitish yellow; the tubules of the distiphallus have black subapical circle in lateral view (Černý 2010: fig. 45).

#### ﻿ Cerodontha (Dizygomyza) tumefacta sp. nov.

Taxon classificationAnimaliaDipteraAgromyzidae

F20C3A71-A34E-5A60-9521-6550346409DC

https://zoobank.org/173CDE77-0AFA-49B1-89BB-94C6C9D02605

Figs 10A–L, 11A–K

##### Type material.

China, Qinghai Province: ***Holotype***: • ♂ (IMAU), Qilian County, Ebu Town, the southern slope of Longkongdaban, 3445 m, 38°04'29.18"N, 100°38'41.05"E, 31.VII.2021, leg. Li Shi. ***Paratypes***: • 6♂♂3♀♀ (IMAU), same data as for holotype; • 2♂♂ (IMAU), Qilian County, Lujiaogou, 3417 m, 38°06'31.22"N, 100°28'42.33"E, 01.VIII.2021, leg. Li Shi. DNA sequence number PX103171 from GenBank.

##### Diagnosis.

Frons and parafacial strongly projecting above eye in lateral view. Fronto-orbital plate obviously shining and inner margin yellow, three (rarely 4 or 5) *ori* and two *ors.* Lunule broad, wider than high and distinctly projecting above frons in lateral view. Mesonotum moderately shiny, 1+3 or 2+3 *dc*, *acr* in four irregular rows. Legs black, fore femora with knees dark yellow. Wing with the ultimate and penultimate sections of M_4_ in proportion of 1.2:1. Calypter yellow, margin and fringe brownish. The mesophallus and distiphallus wrapped in a layer of membrane with dark dots; distiphallus S-shaped, 1.8 × length of mesophallus, pale brown at apex in lateral view.

##### Description.

**Figure 10.**
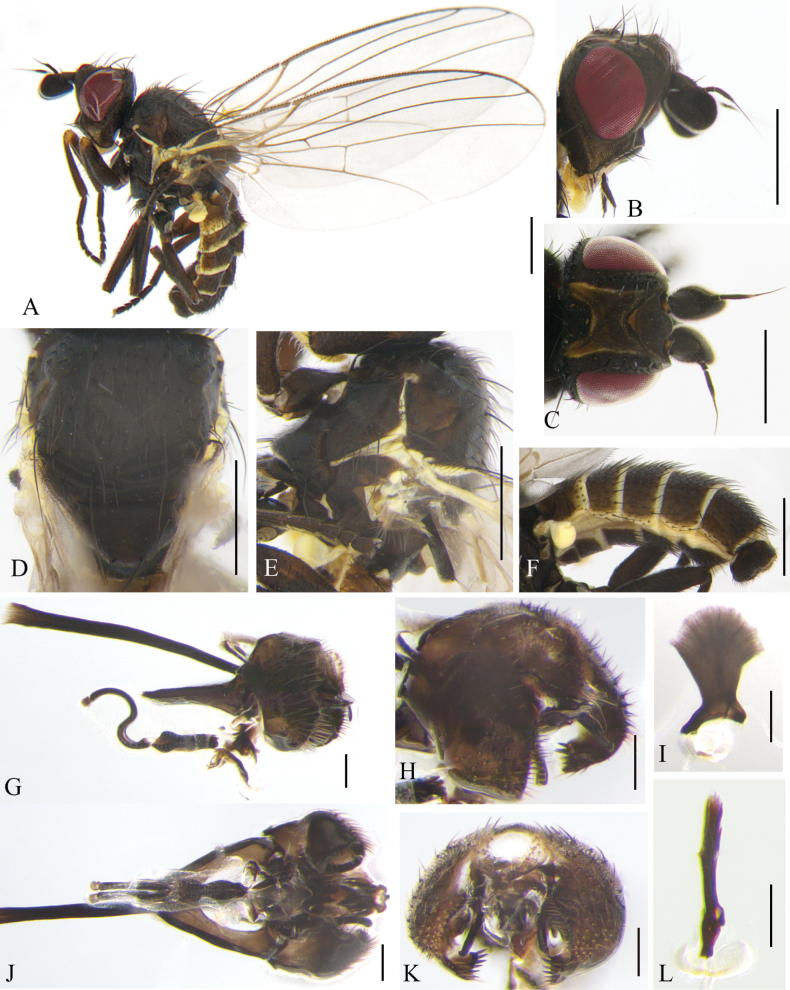
Cerodontha (Dizygomyza) tumefacta sp. nov. Paratype male. **A.** Habitus, lateral view; **B, C.** Head, lateral and dorsal view; **D, E.** Thorax, dorsal and lateral view; **F.** Abdomen, lateral view; **G, J.** Genitalia, lateral and ventral view; **H, K.** Epandrial complex, posterolateral and posterior view; **I, L.** Ejaculatory apodeme, lateral and ventral view. Scale bars: 0.5 mm (**A–F**); 0.1 mm (**G–L**).

**Figure 11.**
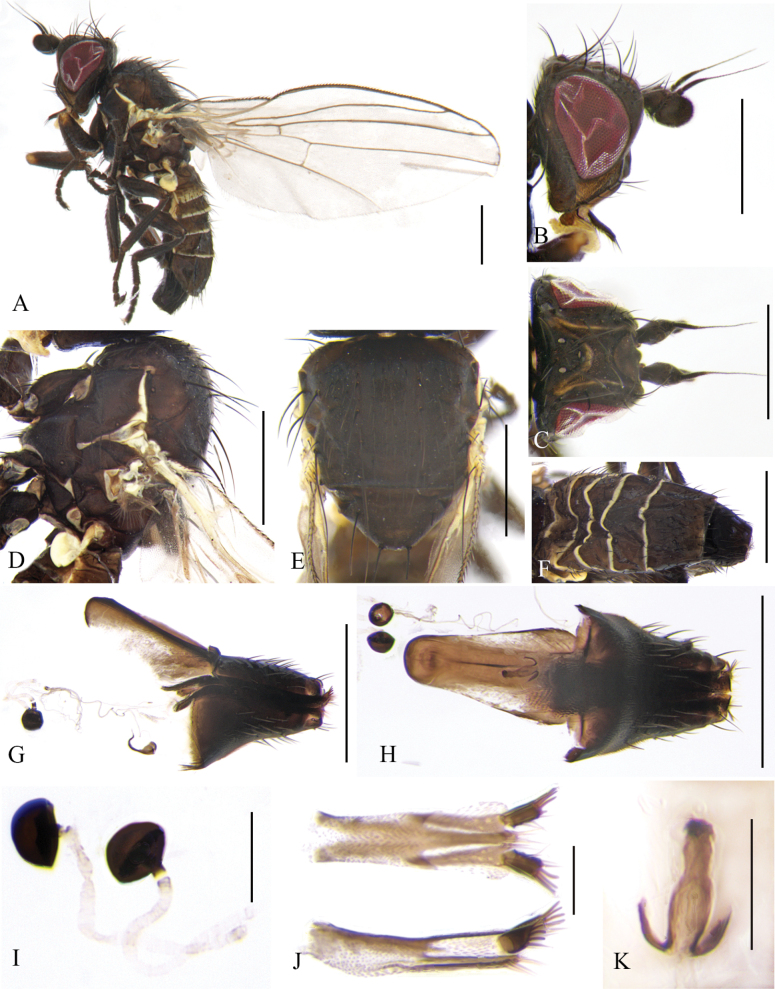
Cerodontha (Dizygomyza) tumefacta sp. nov. Paratype female. **A.** Habitus, lateral view; **B, C.** Head, lateral and dorsal view; **D, E.** Thorax, lateral and dorsal view; **F.** Abdomen, dorsal view; **G, H.** Spermathecae, ventral receptacle and oviscape, lateral and ventral view; **I.** Spermathecae; **J.** Proctiger, lateral and ventral view; **K.** Ventral receptacle, ventral view. Scale bars: 0.5 mm (**A–H**); 0.1 mm (**I–K**).

**Male** (Fig. 10A–L). Body length 2.5–3.0 mm; wing length 2.4–2.7 mm. Female (Fig. 11A–K). Body length 2.3–2.7 mm; wing length 2.3–2.8 mm.

Head (Fig. 10B, C) blackish brown. Frons and parafacial strongly projecting above eye in lateral view; frons ~1.7 × as wide as eye in dorsal view; fronto-orbital plate obviously shiny and inner margin yellow, ~2/5 width of frons; three (rarely 4 or 5) *ori* and two *ors*; orbital setulae sparse and long in one or two rows, reclinate or erect. Ocellar triangle wide, ocellar setae slightly weaker than *ors.* Lunule broad, wider than high and distinctly projecting above frons in lateral view. Antennal first flagellomere very broad with pale pubescence; arista distinctly thickened on basal 1/2. Facial keel wide and distinctly raised. Gena slightly yellow at middle, ~1/3 height of eye, highest point located at the rear. Clypeus yellowish; palpus black, with two prominent setae at apex.

Thorax (Fig. 10D, E) black. Mesonotum moderately shiny, 1+3 or 2+3 *dc*, *acr* in four irregular rows; one postsutural intra-alar, one presutural and two strong postsutural supra-alar setae. Notopleuron brown. Anepisternum with one anepisternal seta, seven long setulae and six short setulae. Katepisternum with one katepisternal seta and five long setulae. Legs black, fore femora with knees dark yellow. Wing: Costa with 2^nd^ (between R_1_ and R_2+3_), 3^rd^ (between R_2+3_ and R_4+5_) and 4^th^ (between R_4+5_ and M_1_) sections in proportion of 4.4:1.3:1; ultimate and penultimate sections of M_1_ in proportion of 2.2:1; *r-m* beyond middle of discal cell; ultimate and penultimate sections of M_4_ in proportion of 1.2:1. Calypter yellow, margin and fringe brownish. Halter yellow.

Abdomen (Fig. 10F) dark brown, tergites 1–6 with yellow posterior margin. Genitalia (Fig. 10G–L): epandrium with a pair of distinctly long claviform processes; surstylus with six spines in posterior view (Fig. 10K); mesophallus and distiphallus wrapped in a layer of membrane with dark dots; mesophallus cylindrical and swollen at distal 1/3, ~1.5 × length of hypophallus; distiphallus S-shaped, 1.8 × length of mesophallus, pale brown at apex in lateral view; ejaculatory apodeme broad, blade margin unclear and sperm pump pale but base of duct lightly pigmented.

**Female** (Fig. 11A–K). Lunule and facial keel narrower than that in male. First flagellomere not enlarged as the male (Fig. 11B). Other external characteristics same as the male except for the female terminalia. Terminalia (Fig. 11G–K): spermathecae circular and truncated at basal 1/5; ventral receptacle symmetrical, well-sclerotized at apex. Cercus with relatively long and sturdy setulae.

##### Distribution.

China (Qinghai).

##### Etymology.

The specific name of this new species comes from the Latin *tumefacta*, meaning intumescent, referring to the lunule projected prominently above the frons.

##### Remarks.

The new species can be distinguished from other species of subgenus Dizygomyza by the lunule projecting prominently above frons and the first flagellomere very broad in male. The mesophallus and distiphallus of Cerodontha (Dizygomyza) tumefacta sp. nov. are similar to those of C. (D.) labradorensis from Palaearctic and Nearctic regions, but it can be separated from the latter by the mesophallus and distiphallus being wrapped in a layer of membrane with dark dots; the mesophallus being cylindrical and swollen at distal 1/3, ~1.5 × length of hypophallus; the distiphallus being S-shaped, 1.8 × length of mesophallus and pale brown at apex. In C. (D.) labradorensis, the mesophallus is swollen at distal 1/3 with a medial suture in ventral view; the distiphallus is S-shaped, ~2.5 × length of mesophallus, and yellowish brown at the apex in lateral view.

### ﻿Subgenus Icteromyza Hendel, 1931

#### ﻿ Cerodontha (Icteromyza) geniculata

Taxon classificationAnimaliaDipteraAgromyzidae

(Fallén, 1823)

809EEF18-9EB9-5CCE-9F5D-047710F80870

Figs 12A–O, 13A–J

Agromyza geniculata Fallén, 1823: 6.
Agromyza flavogeniculata Roser, 1840: 63; Hendel 1931–1936: 54.
Dizygomyza geniculata : Hendel 1920: 132; Hering 1927: 41.
Dizygomyza (Icteromyza) geniculata : Hendel 1931–1936: 55
Dizygomyza (Poemyza) lunzensis Hering in Lindner 1943: 246; Zlobin 2000: 56.
Phytobia (Icteromyza) geniculata : Frick 1952: 392; Spencer 1963: 329.
Icteromyza geniculata : Nowakowski 1962: 99.
Cerodontha (Icteromyza) geniculata : Nowakowski 1967: 655; 1972: 737; 1973: 38; Spencer 1976: 173; Soós and Papp 1984: 284; Sasakawa and Fan 1985: 281; Dursun et al. 2015: 163; Černý et al. 2018: 25; Zlobin 2000: 56; Doukale Daief et al. 2025: 88.

##### Specimens examined.

China, Inner Mongolia, Genhe City, Mangui Town, the northern primitive forest region of Greater Khingan Mountains, Aba River Third Branch Line: • 1♂ (IMAU), unburned area, Pinus sylvestris var. mongholica, malaise trap 33 (three meters above the ground), 52°22'00.00"N, 121°27'07.00"E, 659 m, 28.VII.2022, leg. Rui Ma, Qin-Jianrong Liu; • 1♂ (IMAU), unburned area, Pinus sylvestris var. mongholica, malaise trap 34 (one meter above the ground), 52°22'00.00"N, 120°27'07.00"E, 659 m, 28.VII.2022, leg. Rui Ma, Qin-Jianrong Liu; • 2♀ (IMAU), unburned area, *Larix gmelinii*, malaise trap 30 (one meter above the ground), 52°18'40.00"N, 121°22'48.60"E, 629 m, 28.VII.2022, leg. Rui Ma, Qin-Jianrong Liu; • 1♂ (IMAU), burned area in 2018, Pinus sylvestris var. mongholica, malaise trap 35, 52°21'07.00"N, 121°27'05.30"E, 643.3 m, 14.VII.2022, leg. Li Shi, Zhi-Wei Wang, Rui Ma. DNA sequence number PX103172 from GenBank.

##### Diagnosis.

**Figure 12.**
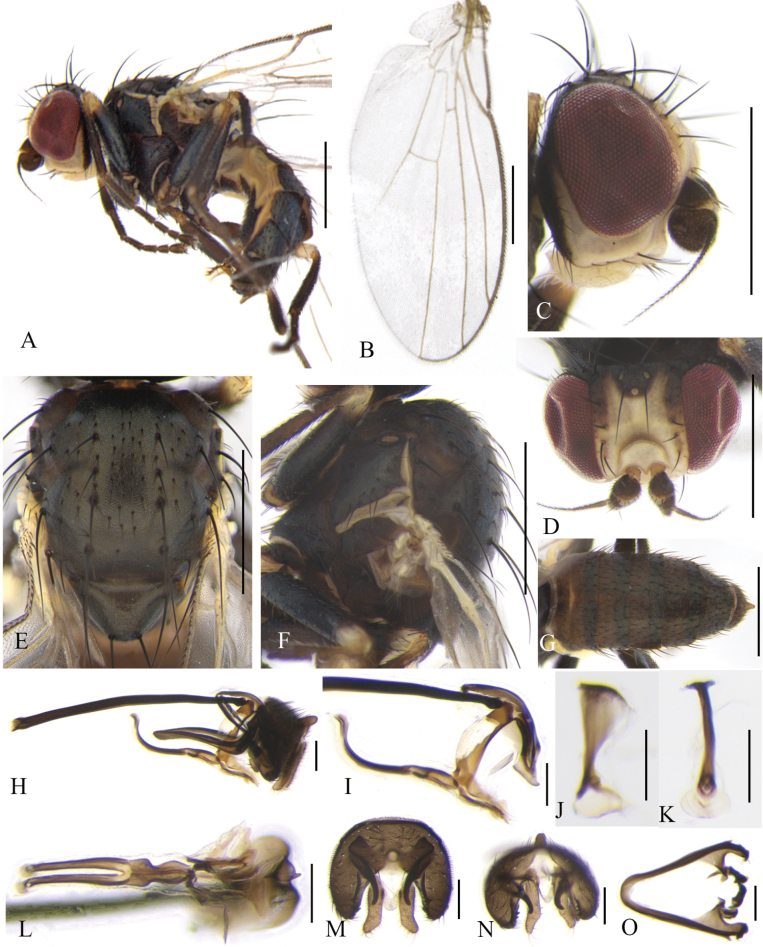
Cerodontha (Icteromyza) geniculata (Fallén, 1823). Male. **A.** Habitus, lateral view; **B.** Wing; **C, D.** Head, lateral and dorsal view; **E, F.** Thorax, dorsal and lateral view; **G.** Abdomen, dorsal view; **H.** Genitalia, lateral view; **I, L.** Phallic complex, lateral and ventral view; **J, K.** Ejaculatory apodeme, lateral and ventral view; **M, N.** Epandrial complex, anterior and anteroventral view; **O.** Hypandrium, ventral view. Scale bars: 0.5 mm (**A–G**); 0.1 mm (**H–O**).

**Figure 13.**
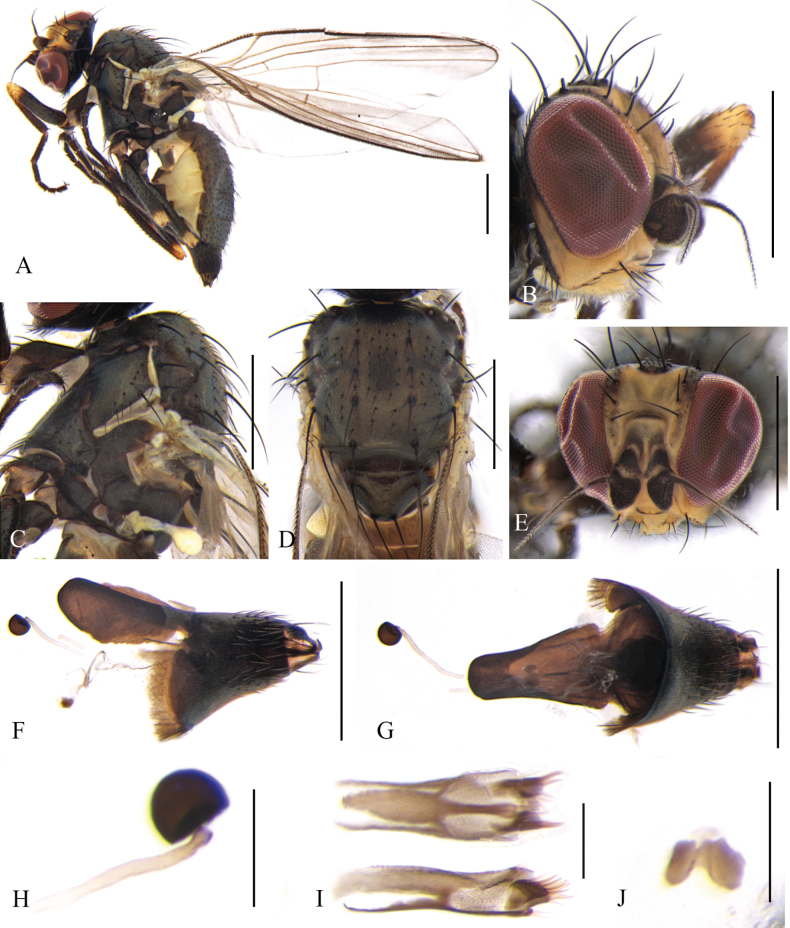
Cerodontha (Icteromyza) geniculata (Fallén, 1823). Female. **A.** Habitus, lateral view; **B, E.** Head, lateral and dorsal view; **C, D.** Thorax, lateral and dorsal view; **F, G.** Spermatheca, ventral receptacle and oviscape, lateral and ventral view; **H.** Spermatheca; **I.** Proctiger, lateral and ventral view; **J.** Ventral receptacle, ventral view. Scale bars: 0.5 mm (**A–G**); 0.1 mm (**H–J**).

Male (Fig. 12A–O). Body length 2.0–2.2 mm; wing length 2.2–2.3 mm. Female (Fig. 13A–J). Body length 1.8–2.3 mm; wing length 2.3–2.7 mm. Frons yellow. Fronto-orbital plate yellow but darkened in upper half, not projecting above eye in lateral view (Fig. 12C, D). Mesonotum entirely black with slightly metallic coloration; 1+3 *dc. acr* in four irregular rows, but two rows beyond level of second *dc* (Fig. 12E, F). Legs black but all knees yellow. Calypter yellow, margin and fringe dark brown. Wing with ultimate and penultimate sections of M_4_ in proportion of 1:1. Male genitalia (Fig. 12H–O): surstylus with three or four spines on inner margin; mesophallus and distiphallus wrapped in a layer of membrane in ventral view. Female terminalia (Fig. 13F–J): spermathecae circular and truncated at basal 1/4; duct slightly sclerotized.

##### Distribution.

Palaearctic: Afghanistan, Albania, Austria, Belgium, Bulgaria, China (Heilongjiang, Inner Mongolia*), Croatia, Czech Republic, Denmark, Estonia, Finland, France, Germany, Greece, Hungary, Iran, Israel, Italy, Japan, Kyrgyzstan, Latvia, Lithuania, Mongolia, Morocco (Doukale Daief et al. 2025), Netherlands, Poland, Portugal (Černý et al. 2018), Romania, Russia, Slovakia, Spain, Sweden, Switzerland, Syria (Soós and Papp 1984), The United Kingdom, Turkey (Dursun et al. 2015), Ukraine, Uzbekistan, Tajikistan, Tunisia (Soós and Papp 1984), Turkmenistan; Oriental: China (Taiwan), India; Afrotropical: South Africa.

##### Remarks.

For more information about this species, refer to Spencer (1976), Nowakowski (1962, 1973), Zlobin (2000), and Černý (2010).

### ﻿Subgenus Poemyza Hendel, 1931

#### ﻿ Cerodontha (Poemyza) beigerae

Taxon classificationAnimaliaDipteraAgromyzidae

Nowakowski, 1972

21B5427F-D0D9-541B-9D98-71814862DF5A

Figs 14A–O, 15A–K

Cerodontha (Poemyza) beigeri Nowakowski, 1972: 742 (holotype and paratype).
Cerodontha (Poemyza) beigerae : Nowakowski 1973: 87; Zlobin 1986: 85; 1993a: 121; 2005, 179; Spencer 1990: 367; Černý and van Zuijlen 2022: 86.

##### Specimens examined.

China, Inner Mongolia, Genhe City, Mangui Town, the northern primitive forest region of Greater Khingan Mountains: • 2♂♂ (IMAU), Wulonggan forestry center at 4217 meters, unburned area, *Larix gmelinii*, malaise trap 13 (three meters above the ground), 52°47'05.26"N, 120°53'40.10"E, 789 m, 28.VII.2022, leg. Rui Ma, Qin-Jianrong Liu; • 2♂♂ (IMAU), Wulonggan forestry center near impounding reservoir, unburned area, Pinus sylvestris var. mongholica, malaise trap 19 (three meters above the ground), 52°47'47.98"N, 120°55'03.45"E, 816 m, 28.VII.2022, Rui Ma, Qin-Jianrong Liu; • 3♂♂ (IMAU), Wulonggan Forestry center near impounding reservoir, unburned area, Pinus sylvestris var. mongholica, Malaise trap 20 (one meter above the ground), 52°47'47.98"N, 120°55'03.45"E, 816 m, 13.VII.2022, leg. Li Shi, Zhi-Wei Wang, Rui Ma; • 1♂ (IMAU), Wulonggan forestry center at 4207 meters, burned area in 2007, Pinus sylvestris var. mongholica, malaise trap 17 (three meters above the ground), 52°51'53.10"N, 120°58'55.80"E,823 m, 28.VII.2022, leg. Rui Ma, Qin-Jianrong Liu; • 1♂ (IMAU), Changliangbeishan houdu, burned area in 2002, Pinus sylvestris var. mongholica, malaise trap 6 (one meter above the ground), 52°26'01.00"N, 120°56'53.20"E, 985 m, 14.VII.2022, leg. Li Shi, Zhi-Wei Wang, Rui Ma; • 1♂ (IMAU), Aba River First Branch Line, unburned area, *Betula platyphylla*, malaise trap 38 (one meter above the ground), 52°12'20.10"N, 120°29'04.90"E, 919 m, 29.VII.2022, leg. Rui Ma, Qin-Jianrong Liu; • 1♀ (IMAU), Yanling Road, burned area in 2007, *Pinus pumila*, malaise trap 22 (one meter above the ground), 28.VII.2022, leg. Rui Ma, Qin-Jianrong Liu; • 1♂ (IMAU), Wulonggan forestry center near impounding reservoir, unburned area, Pinus sylvestris var. mongholica, malaise trap 20 (one meter above the ground), 52°47'47.98"N, 120°55'03.45"E, 816 m, VII.2022, leg. Rui Ma, Qin-Jianrong Liu; • 1♀ (IMAU), Changliangbeishan houdu, burned area in 2002, *Larix gmelinii*, malaise trap 2 (one meter above the ground), 52°25'31.24"N, 120°54'15.76"E, 1032 m, 28.VII.2022, leg. Rui Ma, Qin-Jianrong Liu; • 1♂ (IMAU), Aba River First Branch Line at the end of the Tower Road, unburned area, Pinus sylvestris var. mongholica, malaise trap 40 (one meter above the ground), 52°13'23.30"N, 121°22'46.80"E, 742 m, 29.VII.2022, leg. Rui Ma, Qin-Jianrong Liu; • 1♂ (IMAU), Aba River First Branch Line at the end of the Tower Road, unburned area, Pinus sylvestris var. mongholica, malaise trap 39 (three meters above the ground), 52°13'23.30"N, 121°22'46.80"E, 742 m, 14.VII.2022, leg. Li Shi, Zhi-Wei Wang, Rui Ma; • 1♂ (IMAU), Aba River Third Branch Line, unburned area, *Larix gmelinii*, malaise trap 29 (three meters above the ground), 52°18'40.00"N, 121°22'48.60"E, 629 m, 28.VII.2022, leg. Rui Ma, Qin-Jianrong Liu; • 1♂1♀ (IMAU), Aba River Third Branch Line, burned area in 2018, Pinus sylvestris var. mongholica, malaise trap 36 (one meter above the ground), 52°21'07.00"N, 121°27'05.30"E, 646.3 m, 28.VII.2022, leg. Rui Ma, Qin-Jianrong Liu; • 1♂ (IMAU), Aba River Third Branch Line, burned area in 2018, *Larix gmelinii*, malaise trap 32 (one meter above the ground), 52°19'54.70"N, 121°25'38.40"E, 615 m, 14.VII.2022, leg. Li Shi, Zhi-Wei Wang, Rui Ma; • 2♂♂ (IMAU), Aba River Third Branch Line, burned area in 2018, *Larix gmelinii*, malaise trap 31 (three meters above the ground), 52°19'54.70"N, 121°25'38.40"E, 651 m, 28.VII.2022, leg. Rui Ma, Qin-Jianrong Liu. DNA sequence number PX103173 from GenBank.

##### Diagnosis.

Frons not projecting above eye in lateral view, ~0.8 × as wide as eye and not parallel-sided in dorsal view. Fronto-orbital plate moderately shiny and inner margin yellow, ~1/2 width of frons; two *ori* inclinate and two *ors* reclinate. Lunule yellowish brown, the width equal to the height. Mesonotum with 1+3 *dc* and *acr* in six irregular rows. Legs dark brown, all femora yellow at distal 1/6. Wing with ultimate and penultimate sections of M_4_ in proportion of 1.4:1. Calypter yellow, margin and fringe yellow. Mesophallus oval at apex and swollen on apical 2/5; distiphallus curved conspicuously and fused to mesophallus at base, with tubules dilated slightly at distal 1/3.

##### Redescription.

**Figure 14.**
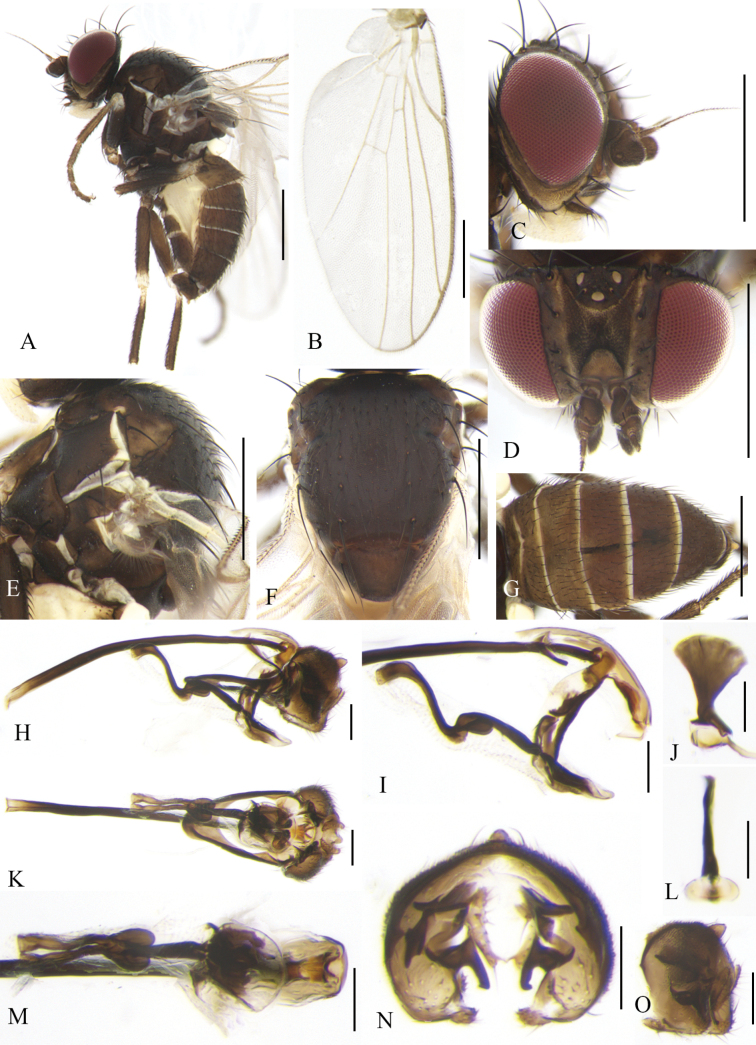
Cerodontha (Poemyza) beigerae Nowakowski, 1972. Male. **A.** Habitus, lateral view; **B.** Wing; **C, D.** Head, lateral and dorsal view; **E, F.** Thorax, lateral and dorsal view; **G.** Abdomen, dorsal view; **H, K.** Genitalia, lateral and ventral view; **I, M.** Phallic complex, lateral and ventral view; **J, L.** Ejaculatory apodeme, lateral and dorsal view; **N, O.** Epandrial complex, anterior and lateral view. Scale bars: 0.5 mm (**A–G**); 0.1 mm (**H–O**).

**Figure 15.**
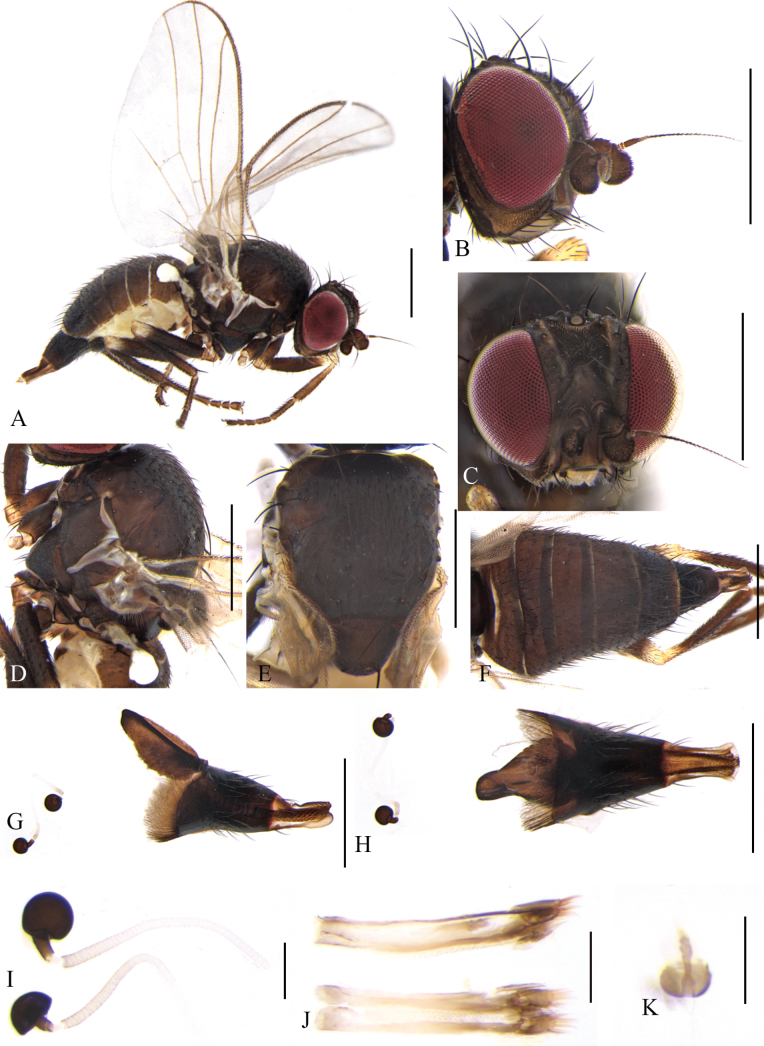
Cerodontha (Poemyza) beigerae Nowakowski, 1972. Female. **A.** Habitus, lateral view; **B, C.** Head, lateral and dorsal view; **D, E.** Thorax, lateral and dorsal view; **F.** Abdomen, dorsal view; **G, H.** Spermathecae, ventral receptacle and oviscape, lateral and ventral view; **I.** Spermathecae; **J.** Proctiger, lateral and ventral view; **K.** Ventral receptacle, ventral view. Scale bars: 0.5 mm (**A–H**); 0.1 mm (**I–K**).

**Male** (Fig. 14A–O). Body length 1.7–1.9 mm; Wing length 1.9–2.3 mm. Female (Fig. 15A–K). Body length 2.0–2.3 mm; Wing length 2.2–2.4 mm.

Head (Fig. 14C, D) brown. Frons not projecting above eye in lateral view, ~0.8 × as wide as eye, not parallel-sided in dorsal view; fronto-orbital plate moderately shiny and inner margin yellow, ~1/2 width of frons; two *ori* inclinate and two *ors* reclinate; orbital setulae reclinate or erect in a single row; inner vertical setae surrounded by yellow coloration; outer vertical setae on brown background. Ocellar triangle yellow on anterior margin, ocellar setae and posterior *ors* equal in length. Lunule yellowish brown, the width equal to the height. Antennal first flagellomere ovate; arista with microscopic pubescence. Gena brownish yellow, ventral edge black, and ~1/5 height of eye. Clypeus yellow; palpus brown with three setae at apex.

Thorax matt black (Fig. 14E, F). Mesonotum with 1+3 *dc*, *acr* in six irregular rows; two postsutural intra-alar, one presutural and two postsutural supra-alar setae. Notopleuron yellowish brown. Anepisternum pale yellow on dorsal and posterior margin, with one strong anepisternal seta and eight short setulae. Katepisternum with one strong seta and four short setulae. Scutellum pale brown in the middle in dorsal view. Legs dark brown, all femora yellow at distal 1/6. Wing: Costa with 2^nd^ (between R_1_ and R_2+3_), 3^rd^ (between R_2+3_ and R_4+5_) and 4^th^ (between R_4+5_ and M_1_) sections in proportion of 4.0:1.6:1; ultimate and penultimate sections of M_1_ in proportion of 2.5:1; *r-m* at middle of discal cell; ultimate and penultimate sections of M_4_ in proportion of 1.4:1. Calypter yellow, margin and fringe yellow. Halter yellowish white.

Abdomen (Fig. 14G) brown, tergites 1–6 with narrow yellow posterior margin. Genitalia (Fig. 14H–O): epandrium with a pair of distinctly claviform processes; surstylus with three or four short setae on inner margin; mesophallus oval at apex and swollen on apical 2/5; distiphallus curved conspicuously and fused to mesophallus at base, with tubules dilated slightly at distal 1/3; ejaculatory apodeme narrow, blade margin clear, sperm pump pale but base of duct lightly pigmented.

**Female.** Notopleuron brown. Abdominal tergites 1–6 with especially narrow yellow posterior margin (Fig. 15F). Genitalia (Fig. 15G–K): two spermathecae unequal in size and circular and truncated at basal 1/5; spermathecae ducts long and curved basally; ventral receptacle symmetrical, oval-shaped at the bottom in ventral view; proctiger slightly sclerotized. Cercus with fine apical setulae.

##### Distribution.

Palaearctic: Belgium, China (Inner Mongolia)^#^, Czech Republic, Germany, Hungary, Mongolia, North Korea (Černý 2007), Netherlands (Černý and van Zuijlen 2022), Poland, Russia, Slovakia, Sweden, Switzerland.

##### Remarks.

This species is very similar to C. (P.) imbuta from the Palaearctic region in the following characteristics: frons dark brown, not projecting above eye; fronto-orbital plate broadening anteriorly; lunule high and narrow; anepisternum black except dorsal margin yellow; legs dark brown with all knees yellow. But it can be differentiated in that the calypter margin and fringe are yellow, the distiphallus has the tubules dilated slightly at distal 1/3 and more curved than that of C. (P.) imbuta. In C. (P.) imbuta, the calypter is yellow with the margin and fringe are brownish black; the tubules of the distiphallus are dilated at distal 1/4 and slightly constricted in the middle (Zlobin 1993a: fig. 55; Nowakowski 1973: fig. 124).

#### ﻿ Cerodontha (Poemyza) muscina

Taxon classificationAnimaliaDipteraAgromyzidae

(Meigen, 1830)

0764C7E4-C448-51A9-A44B-AE6931C119B9

Figs 16A–N, 17A–K

Agromyza muscina Meigen, 1830: 177.
Agromyza marginata Loew, 1869: 49. Frick 1957: 202.
Dizygomyza (*Poëmyza*) muscina: Hendel 1931–1936: 44.
Phytobia (*Poëmyza*) muscina: Frick 1952: 392, 1957: 202.
Cerodontha (Poemyza) muscina : Nowakowski 1967: 649, 1973: 104; Spencer 1969: 132; Soós and Papp 1984: 289; Spencer and Steyskal 1986: 93; Scheffer et al. 2007: 771; Guglya 2021: 39; Lonsdale 2021: 296; Černý et al. 2022: 417.
Cerodontha muscina : Boucher and Wheeler 2001: 614.

##### Specimens examined.

China, Inner Mongolia, Genhe City, Mangui Town, the northern primitive forest region of Greater Khingan Mountains: • 4♂♂ (IMAU), Wulonggan forestry center near impounding reservoir, unburned area, Pinus sylvestris var. mongholica, malaise trap 20 (one meter above the ground), 52°47'47.98"N, 120°55'03.45"E, 816 m, 13.VII.2022, leg. Li Shi, Zhi-Wei Wang, Rui Ma; • 1♀ (IMAU), Changliangbeishan houdu, burned area in 2002, Pinus sylvestris var. mongholica, malaise trap 4 (one meter above the ground), 52°25'31.24"N, 120°54'40.10"E, 1009 m, 28.VII.2022, leg. Rui Ma, Qin-Jianrong Liu; • 1♂ (IMAU), Aba River Third Branch Line, unburned area, Pinus sylvestris var. mongholica, malaise trap 33 (three meters above the ground), 52°22'00.00"N, 121°27'07.00"E, 659 m, 28.VII.2022, leg. Rui Ma, Qin-Jianrong Liu; • 1♀ (IMAU), Changliangbeishan at 4268 meters, unburned area, *Larix gmelinii*, malaise trap 7 (three meters above the ground), 52°29'15.18"N, 120°02'24.80"E, 920 m, 09.VII.2022, leg. Rui Ma, Qin-Jianrong Liu; • 1♂1♀ (IMAU), Aba River Third Branch Line, unburned area, *Larix gmelinii*, malaise trap 30 (one meter above the ground), 52°18'40.00"N, 121°22'48.60"E, 629 m, 28.VII.2022, leg. Rui Ma, Qin-Jianrong Liu; • 1♂1♀ (IMAU), Aba River Third Branch Line, burned area in 2018, *Larix gmelinii*, malaise trap 32, 52°19'57.70"N,121°25'38.40"E, 651 m, 28.VII.2022, leg. Rui Ma, Qin-Jianrong Liu; • 1♂ (IMAU), Aba River Third Branch Line, burned area in 2018, Pinus sylvestris var. mongholica, malaise trap 35 (three meters above the ground), 52°21'07.00"N, 121°27'05.30"E, 643.3 m, 14.VII.2022, leg. Li Shi, Zhi-Wei Wang, Rui Ma. DNA sequence number PX102344 from GenBank.

##### Diagnosis.

**Figure 16.**
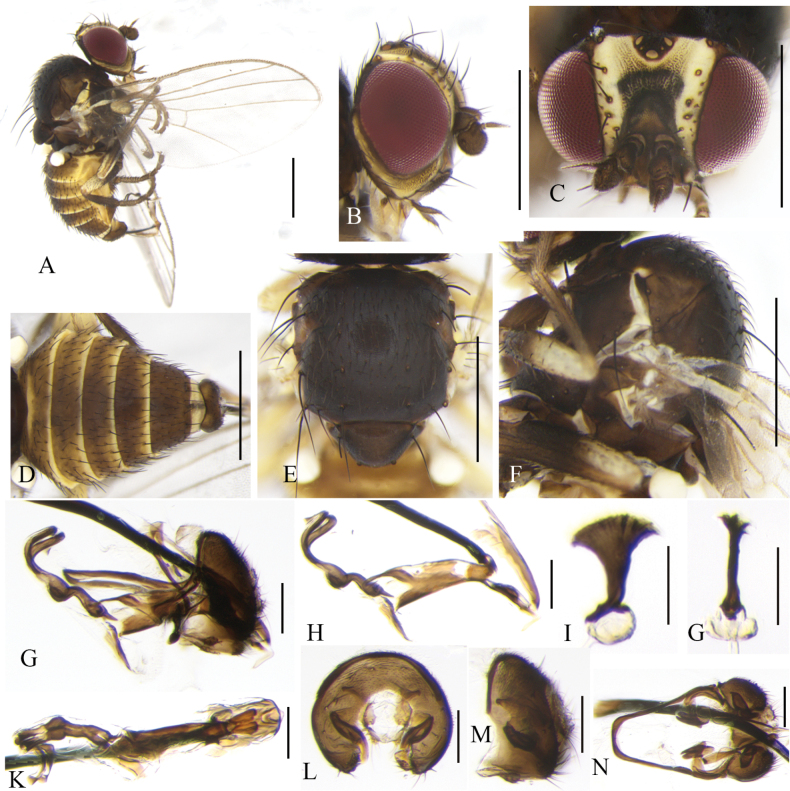
Cerodontha (Poemyza) muscina (Meigen, 1830). Male. **A.** Habitus, lateral view; **B, C.** Head, lateral and dorsal view; **D.** Abdomen, dorsal view; **E, F.** Thorax, dorsal and lateral view; **G.** Genitalia, lateral view; **H, K.** Phallic complex, lateral and ventral view; **I, G.** Ejaculatory apodeme, lateral and ventral view; **L, M.** Epandrial complex, anterior and lateral view. **N.** Hypandrium, ventral view. Scale bars: 0.5 mm (**A–F**); 0.1 mm (**G–N**).

**Figure 17.**
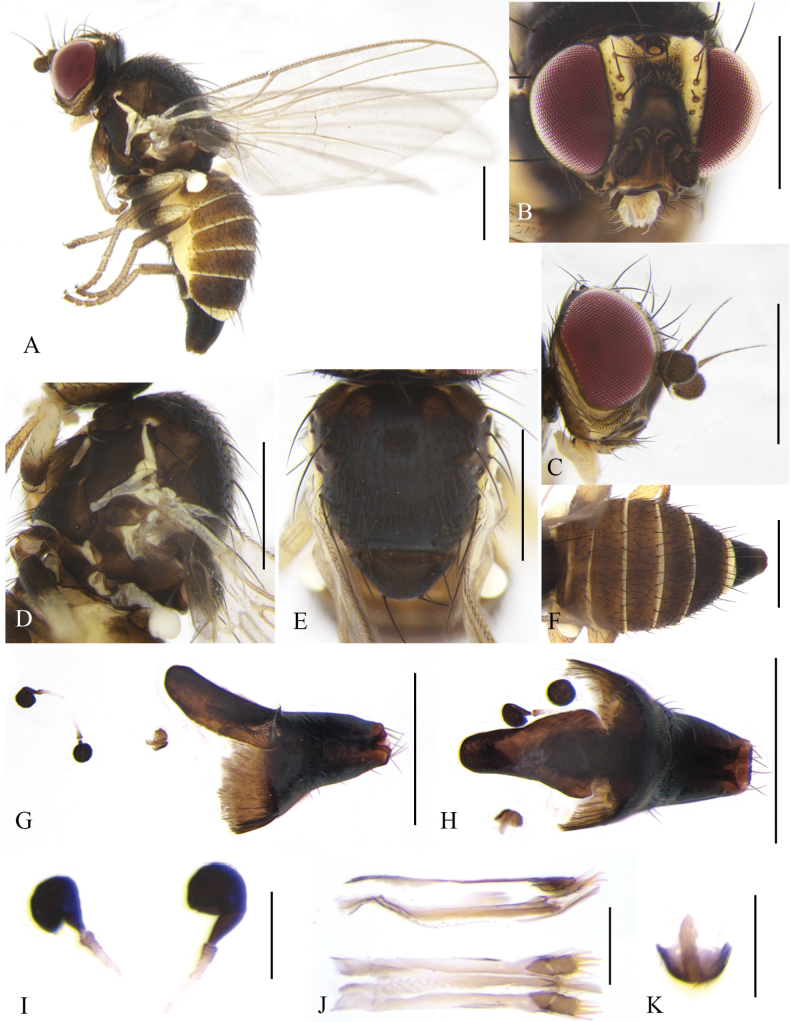
Cerodontha (Poemyza) muscina (Meigen, 1830). Female. **A.** Habitus, lateral view; **B, C.** Head, anterior and lateral view; **D, E.** Thorax, lateral and dorsal view; **F.** Abdomen, dorsal view; **G, H.** Spermathecae, ventral receptacle and oviscape, lateral and ventral view; I spermathecae; **J.** Proctiger, lateral and ventral view; **K.** Ventral receptacle, ventral view. Scale bars: 0.5 mm (**A–H**); 0.1 mm (**I–K**).

Male (Fig. 16A–N). Body length 1.7–2.0 mm; wing length 1.7–2.1 mm. Female (Fig. 17A–K). Body length 1.9–2.1 mm; wing length 1.8–2.1 mm (Lonsdale 2021: male wing length 1.7–2.5 mm, female wing length 1.4–2.3 mm). Frons mostly yellow with black transverse line at middle. Fronto-orbital plate yellow, ~1/2 width of frons, and orbital setae surrounded by a brown circle (Fig. 16C). Two *ori* inclinate and two *ors* reclinate. Lunule dark brown, higher than wide. Mesonotum with 0-1+3 *dc*, anteriormost postsutural *dc* close to suture; *acr* in six rows (Fig. 16E). Legs dark brown, all femora yellow at distal 1/3–1/2. Wing with ultimate and penultimate sections of M_4_ in proportion of 1.1:1. Male genitalia (Fig. 16G–N): distiphallus (Fig. 16H, K) with tubules short, mightily contorted to right, relatively dilated at apex. Female terminalia (Fig. 17G–K): spermathecae (Fig. 17I) with semicircular and rough-surfaced on apical half, and subconical and slightly curved on basal half.

##### Distribution.

Palaearctic: Andorra, Austria (Soós and Papp 1984), Belarus, Belgium, Bulgaria (Černý et al. 2022), China (Inner Mongolia)^#^, Czech Republic, Denmark, Estonia, Finland, France (Soós and Papp 1984), Germany, Greece, Hungary, Italy, Kyrgyzstan, Latvia, Lithuania, Netherlands, Norway, Poland, Portugal (Černý et al. 2018), Russia, Slovakia, South Korea, Spain, Sweden, Switzerland, The United Kingdom, Turkey (Dursun et al. 2015), Ukraine (Guglya 2021), The Former Yugoslavia (Soós and Papp 1984); Nearctic: Canada, USA (Lonsdale 2021); Afrotropical: Madagascar (https://www.gbif.org/species/1552471).

##### Remarks.

For more information about this species, refer to Lonsdale (2021) and Guglya (2021).

###### ﻿Key to the known species of the genus *Cerodontha* in China

(Referenced from Spencer 1987; Boucher 2010; Lonsdale and von Tschirnhaus 2021).

**Table:** 

1	First flagellomere with short spine or projection at anterodistal corner (Fig. 2C); scutellum only with apical scutellar setae (Fig. 2E) (subgenus Cerodontha)	**2**
–	First flagellomere normal, sometimes enlarged in male (Fig. 6B); scutellum with both lateral and apical scutellar setae	**6**
2	Frons with 1 *ori* and 2 *ors*	**3**
–	Frons with 3–4 *ori* and 2*ors*	**4**
3	Mesonotum shining black, *acr* in 2 rows (Fig. 4D); a slight fracture separated distiphallus from mesophallus, and distiphallus with white subapical circle and tiny brown protrusion pointed apically in lateral view (Fig. 4G)	***C. (C.) fulvipes***
–	Mesonotum matt grayish-black or yellow centrally and adjoining scutellum, *acr* absent; distiphallus confluent with mesophallus, and distiphallus big trumpet-shaped in lateral view (Spencer 1976: fig. 319)	***C. (C.) denticornis***
4	Scutellum yellow in the middle (Fig. 2E); distiphallus gourd-shaped with enlarged apex and complete ventral opening (Fig. 2H, L)	***C. (C.) flavicornis***
–	Scutellum entirely black; distiphallus not as above	**5**
5	Mesonotum with *acr* in irregularly in 4 or 5 rows; wing with ultimate section of M_4_ equal to penultimate section; *r-m* before middle of discal cell; distiphallus half length of mesophallus (Spencer 1976: fig. 315)	***C. (C.) hennigi***
–	Mesonotum with *acr* in 8 rows; wing with ultimate section of M_4_ slightly longer than penultimate section; *r-m* at middle of discal cell; distiphallus > 2 × as long as mesophallus (Sasakawa 1996: fig. 24A, B)	***C. (C.) fujianensis***
6	Lunule distinctly higher than wide (Boucher 2010: figs 6a, b, 7)	**7**
–	Lunule distinctly wider than high (Boucher 2010: fig. 5)	**8**
7	Lunule broad, slightly higher than wide, gradually tapering to a point dorsally, or becoming narrow from the middle to dorsal half and blunt on top (Boucher 2010: fig. 7); mesoscutum with a pair of prescutellar setae; surstylus and sometimes lower margin of epandrium with strong setae or spines (subgenus Butomomyza)	**9**
–	Lunule narrow, higher than wide, sometimes sunken beneath prominent orbital plates (Boucher 2010: fig. 6a, b); mesoscutum without a pair of prescutellar setae; surstylus usually without strong setae or spines (sometimes with bulges or fine setulae) (subgenus Poemyza)	**10**
8	Head usually with frons, lunule, face, or gena bright yellow; ocellar triangle extended anteriorly beyond level of *ors*; first flagellomere not enlarged in males (subgenus Icteromyza)	**18**
–	Head usually mostly brown or partially yellowish-brown (usually not bright yellow); ocellar triangle shorter, not extended beyond level of *ors*; first flagellomere often enlarged in males (subgenus Dizygomyza)	**24**
9	Legs black, only with fore knees yellow, middle knees narrowly yellowish and all tarsi yellowish brown; distiphallus with distal two thirds of distiphallus bifid (Sasakawa 1963a: fig. 12)	***C. (B.) cornigera***
–	Legs brownish black, all knees yellowish brown; distiphallus not bifid (Chen and Wang 2003: fig. 4)	***C. (B.) fujianica***
10	Frons uniformly brown to black	**11**
–	Frons or fronto-orbital plate at least partially yellow	**14**
11	Mesonotum without presutural dorsocentral seta	**12**
–	Mesonotum with a presutural dorsocentral seta	**13**
12	Abdominal tergite 6 with 2 large yellowish lateral spots in dorsal view (Zlobin 1993a: fig. 71); distiphallus with a pair of short tubules fused at the middle, below the base with a pair of sclerotized plates (Zlobin 1993a: fig. 70)	***C. (P.) oryziphila***
–	Abdominal tergite 6 brownish yellow on posterior margin, but no lateral spots in dorsal view; distiphallus dilated without tubules (Sasakawa 1961: fig. 49)	***C. (P.) bisetiorbita***
13	Legs black, only with fore knees yellow; distiphallus with tubules trumpet-shaped at apex. (Spencer 1976: fig. 343)	***C. (P.) incisa***
–	Legs black, but all femora at distal 1/6 and all knees yellow; distiphallus with tubules dilated slightly at distal 1/3 (Fig. 14I, M)	***C. (P.) beigerae***
14	Notopleuron yellow (Fig. 2D)	**15**
–	Notopleuron brown (Fig. 16F)	**16**
15	Fronto-orbital plate shining black below anterior *ors*; epandrium with a dorsal process in lateral view (Spencer 1976: fig. 338)	***C. (P.) lateralis***
–	Fronto-orbital plate mostly yellow; epandrium without dorsal process (Spencer 1976: fig. 341)	***C. (P.) superciliosa***
16	Wing with *r-m* at middle of discal cell; distiphallus with distal tubules rotated to right (Spencer 1976: fig. 335)	***C. (P.) muscina***
–	Wing with *r-m* distinctly before middle of distal cell; distiphallus not as above	**17**
17	Calypter yellowish, margin and fringe brown; gena very narrow, ~1/11 height of eye; femora black, all knees distinctly yellow; distiphallus with dense ventral setulae and bifurcated at apex in lateral view (Sasakawa 1972: fig. 20)	***C. (P.) hirta***
–	Calypter, margin and fringe yellowish white; gena ~1/5 height of eye; femora black but yellow at least at distal 1/3; distiphallus fused to mesophallus, and distiphallus without ventral setulae (Spencer 1990: fig. 1392)	***C. (P.) setariae***
18	Femora entirely yellow	***C. (I.) piliseta***
–	Femora mostly black and yellow distally	**19**
19	Wing with ultimate section of vein M_4_ shorter than penultimate section	***C. (I.) nigricoxa***
–	Wing with ultimate section of vein M_4_ equal to or longer than penultimate section	**20**
20	Antenna and palpus black; sum of the lengths of mesophallus and distiphallus ~6 × as long as the basiphallus (Sasakawa 2008: fig. 1)	***C. (I.) alishana***
–	Antenna and palpus partially or entirely yellow to dark brown; sum of the lengths of mesophallus and distiphallus ~3–5 × as long as the basiphallus	**21**
21	Fronto-orbital plate entirely brown; distiphallus with a big curve in lateral view	**22**
–	Fronto-orbital plate yellow but brown in upper half; distiphallus more straight (Fig. 12I, L)	**23**
22	Distiphallus with tubules distinctly longer than that in C. (I.) rishi (Spencer 1961: fig. 44)	***C. (I.) duplicata***
–	Distiphallus with C-shaped curve (Garg 1971: fig. 7e)	***C. (I.) rishii***
23	Antennal first flagellomere brown; surstylus with 3 or 4 spines on inner margin	***C. (I.) geniculata***
–	Antennal first flagellomere yellow; surstylus with 10 spines on inner margin	***C. (I.) taipingensis***
24	Legs black with all knees bright yellow	**25**
–	Legs yellow at least with hind knees black	**26**
25	Lunule yellow with variable brown (Fig. 6C, D); mesonotum with a pair of prescutellar setae; tubules of distiphallus with deep basal curve and not recurved apically (Fig. 6I)	***C. (D.) flavilunulata* sp. nov.**
–	Lunule velvety grayish; prescutellar setae absent; distiphallus with shallow basal curve but more recurved apically (Lonsdale 2021: figs 533, 534)	***C. (D.) morosa***
26	Abdominal tergites entirely black without narrow yellow posterior margin	**27**
–	Abdominal tergites with 1–6 yellowish posterior margin (Fig. 9F)	**29**
27	Mesoscutum with a pair of prescutellar setae; calypter margin and fringe light yellow; distiphallus thin, conspicuously longer than mesophallus (Spencer 1976: fig. 379)	***C. (D.) caricicola***
–	Mesoscutum without prescutellar setae; calypter yellowish, margin or fringe brown; distiphallus not as above	**28**
28	Mid tibia with one posterodorsal seta; wing with ultimate section of M_4_ shorter than penultimate section	***C. (D.) vietnamensis***
–	Mid tibia without posterodorsal seta; wing with ultimate section of M_4_ equal to or slightly longer than penultimate section	***C. (D.) omissa***
29	Calypter, margin and fringe whitish yellow; tubules of the distiphallus with black subapical circle in lateral view (Nowakowski 1973: fig. 165; Černý 2010: fig. 45)	***C. (D.) luctuosa***
–	Calypter yellowish, margin light brown, fringe brown; distiphallus with distal tubules sclerotized uniformly	**30**
30	Lunule and parafacial broad, distinctly projecting above frons (Fig. 10B); gena with the highest point located at the rear (Fig. 11B)	***C. (D.) tumefacta* sp. nov.**
–	Lunule at least not projecting above frons (Fig. 9B); gena with the highest point located in the middle (Fig. 8B)	**31**
31	Frons dark brown in the center; antenna with arista thickened on basal half; gena brownish yellow in the center and dark brown on both sides; distiphallus ~2.5 × longer than mesophallus and yellowish brown at apex in lateral view (Fig. 9K, L); surstylus with 5 thick spines on inner margin (Fig. 9H)	***C. (D.) labradorensis***
–	Frons yellowish brown in the center; antenna with arista thickened on basal 1/4; gena yellowish brown; distiphallus ~1.5 × longer than mesophallus and dark brown at apex in lateral view (Fig. 8H, K); surstylus with ~25 short and thick spines (Fig. 8I)	***C. (D.) granditerga* sp. nov.**

###### ﻿Checklist of thirty-two *Cerodontha* species in China

**1. Cerodontha (Butomomyza) cornigera (Meijere, 1934) (as Dizygomyza (Poemyza))**

**Literature.**Spencer 1961, 1990; Sasakawa 1972; Zlobin 2001c; Chen and Wang 2003.

**Distribution.** Oriental: China (Taiwan), Malaysia, Indonesia.

**2. Cerodontha (Butomomyza) fujianica Chen & Wang, 2003**

**Literature.**Chen and Wang 2003.

**Distribution.** Oriental: China (Fujian).

**3. Cerodontha (Cerodontha) denticornis (Panzer, 1806) (as *Chlorops*)**

*Agromyza acuticornis* Meigen, 1830 (synonym).

*Agromyza confinis* Meigen, 1830 (synonym).

*Agromyza nigritarsis* Meigen, 1830 (synonym).

*Agromyza tarsella* Zetterstedt, 1848 (synonym).

Ceratomyza denticornis var. nigriventris Strobl, 1900 (synonym).

Ceratomyza denticornis var. nigroscutellata Strobl, 1900 (synonym).

*Ceratomyza semivittata* Strobl, 1909 (synonym).

*Cerodontha lacustris* Garg, 1971 (synonym).

*Cerodontha narkandae* Singh & Ipe, 1973 (synonym).

*Cerodontha testae* Singh & Ipe, 1973 (synonym).

**Literature.**Nowakowski 1967, 1973; Spencer 1976; Soós and Papp 1984; Černý et al. 2018.

**Distribution.** Palaearctic: Afghanistan, Albania, Andorra, Austria, Belarus, Belgium, Bulgaria, China (Beijing, Hebei, Inner Mongolia, Xinjiang), Czech Republic, Croatia, Denmark, Finland, France, Estonia, Germany, Greece, Hungary, Ireland, Italy, Japan, Latvia, Lebanon, Lithuania, Malta, Mongolia, Montenegro, Morocco, Netherlands, Norway, Poland, Portugal, Russia, Slovakia, Spain, Sweden, Switzerland, The United Kingdom, Tunisia, Turkey, Ukraine, Uzbekistan (Soós and Papp 1984); Oriental: China (Taiwan), India; Afrotropical: Madagascar (https://www.gbif.org/species/12105471).

**4. Cerodontha (Cerodontha) flavicornis (Egger, 1862) (as *Ceratomyza*)**

**Literature.**Nowakowski 1973; Soós and Papp 1984; Spasić 1996; Černý 2018; Černý and Bächli 2018.

**Distribution.** Palaearctic: Albania, Andorra, Austria, Belgium, China (Qinghai)^#^, Croatia, France, Germany, Hungary, Italy, Montenegro, Poland, Russia, Spain, Switzerland.

**5. Cerodontha (Cerodontha) fujianensis Sasakawa, 1996**

**Literature.**Sasakawa 1996; Zlobin 2001a.

**Distribution.** Oriental: China (Fujian).

**6. Cerodontha (Cerodontha) fulvipes (Meigen, 1830) (as *Agromyza*)**

**Literature.**Sasakawa 1961; Nowakowski 1973; Spencer 1976; Soós and Papp 1984; Spasić 1996; Černý et al. 2018; Černý et al. 2022.

**Distribution.** Palaearctic: Austria, Belgium, Bulgaria, China (Xinjiang, Qinghai*), Croatia, Czech Republic, Denmark, Estonia, Finland, France, Germany, Greece, Hungary, Ireland, Italy, Japan, Latvia, Lithuania, Montenegro, Netherlands, Norway, Poland, Portugal, Romania, Russia, Slovakia, Spain, Sweden, Tajikistan, The United Kingdom, Turkey, Ukraine, Uzbekistan (Soós and Papp 1984).

**7. Cerodontha (Cerodontha) hennigi Nowakowski, 1967**

**Literature.**Nowakowski 1973; Spencer 1976; Soós and Papp 1984

**Distribution.** Palaearctic: Austria, China (Xinjiang), Czech Republic, Denmark, Finland, Germany, Hungary, Italy, Kazakhstan, Russia, Slovakia, Spain, Sweden, The United Kingdom.

**8. Cerodontha (Dizygomyza) caricicola (Hering, 1926) (as *Dizygomyza*)**

*Dizygomyza soenderupi* Hering, 1937 (synonym).

**Literature.**Spencer 1976; Nowakowski 1967, 1973; Soós and Papp 1984; Sasakawa 2005; Černý 2018.

**Distribution.** Palaearctic: Austria, China (Xinjiang), Czech Republic, Denmark, Estonia, France, Germany, Hungary, Japan, Latvia, Lithuania, Mongolia, Norway, Poland, Russia, Slovakia, South Korea, Sweden, Switzerland, The United Kingdom, Turkey.

**9. Cerodontha (Dizygomyza) flavilunulata sp. nov.**

**Distribution.** Palaearctic: China (Inner Mongolia).

**10. Cerodontha (Dizygomyza) granditerga sp. nov.**

**Distribution.** Palaearctic: China (Qinghai).

**11. Cerodontha (Dizygomyza) labradorensis Spencer, 1969**

Cerodontha (Dizygomyza) orbitalis Zlobin, 1984 (synonym).

Cerodontha (Dizygomyza) poolei Spencer, 1986 (synonym).

**Literature.**Spencer 1969; Zlobin 1984b; Spencer and Steyskal 1986.

**Distribution.** Palaearctic: China (Qinghai)^#^, Kirgizia, Mongolia, Russia, Tajikistan, Uzbekistan; Nearctic: Canada, USA.

**12. Cerodontha (Dizygomyza) luctuosa (Meigen, 1830) (as *Agromyza*)**

*Dizygomyza effusi* Karl, 1926 (synonym).

**Literature.**Sasakawa 1961, Nowakowski 1967; Spencer 1976; Boucher 2005; Černý 2010, 2018; Doukale Daief et al. 2025.

**Distribution.** Palaearctic: Alaska, Albania, Austria, Belarus, Belgium, China (Xinjiang), Bulgaria, Belarus, Croatia, Czech Republic, Denmark, Finland, France, Germany, Greece, Hungary, Iraq, Ireland, Israel, Italy, Japan, Kazakhstan, Latvia, Lithuania, Morocco, Netherlands, Norway, Poland, Portugal, Romania, Russia, Slovakia, Spain, Sweden, Switzerland, The United Kingdom, Tunisia, Turkey, Uzbekistan; Nearctic: Canada, USA.

**13. Cerodontha (Dizygomyza) morosa (Meigen, 1830) (as *Agromyza*)**

*Agromyza groddicornis* Zetterstedt, 1860 (synonym).

*Cerodontha graminiphila* Garg, 1971 (synonym).

Cerodontha (Dizygomyza) islandica Griffiths, 1968 (synonym).

**Literature.**Sasakawa 1961; Nowakowski 1973; Spencer 1976; Soós and Papp 1984; Černý 2007.

**Distribution.** Palaearctic: Austria, Bulgaria, Estonia, China (Xinjiang), Czech Republic, Finland, France, Germany, Hungary, Iceland, Ireland, Japan, Latvia, Lithuania, Netherlands, North Korea, Norway, Poland, Russia, Slovakia, Sweden, Switzerland, The United Kingdom, Turkey; Oriental: India; Nearctic: Canada, USA.

**14. Cerodontha (Dizygomyza) omissa (Spencer, 1961) (as *Phytobia*)**

*Phytobia ochreata* Sasakawa, 1963 (synonym).

**Literature.**Spencer 1961, 1966; Sasakawa 1963b, 2005.

**Distribution.** Oriental: China (Taiwan), Indonesia (Lombok), Japan (Ryukyu).

**15. Cerodontha (Dizygomyza) tumefacta sp. nov.**

**Distribution.** Palaearctic: China (Qinghai).

**16. Cerodontha (Dizygomyza) vietnamensis (Sasakawa, 1963) (as Phytobia (Dizygomyza)).**

**Literature.**Sasakawa 2006.

**Distribution.** Oriental: China (Hong Kong), Vietnam.

**17. Cerodontha (Icteromyza) alishana Sasakawa, 2008**

**Literature.**Sasakawa 2008.

**Distribution.** Oriental: China (Taiwan).

**18. Cerodontha (Icteromyza) duplicata (Spencer, 1961) (as *Phytobia*)**

*Cerodontha keethamensis* Garg, 1971 (synonym).

*Cerodontha periyari* Singh & Ipe, 1973 (synonym).

*Phytobia hardyi* Sasakawa, 1963 (synonym).

**Literature.**Spencer 1961, 1986; Sasakawa 1963b, 1977, 2005, 2006.

**Distribution.** Oriental: China (Fujian, Hong Kong, Yunnan, Taiwan), India, Indonesia, Nepal, Philippines, Thailand, Vietnam; Australian: Papua New Guinea (New Britain).

**19. Cerodontha (Icteromyza) geniculata (Fallén, 1823) (as *Agromyza*)**

*Agromyza flavogeniculata* Roser, 1840 (synonym).

Dizygomyza (Poemyza) lunzensis Hering, 1943 (synonym).

**Literature.**Nowakowski 1973; Spencer 1976; Soós and Papp 1984; Zlobin 2000; Dursun et al. 2015; Černý et al. 2018; Doukale Daief et al. 2025.

**Distribution.** Palaearctic: Afghanistan, Albania, Austria, Belgium, Bulgaria, China (Heilongjiang, Inner Mongolia*), Croatia, Czech Republic, Denmark, Estonia, Finland, France, Germany, Greece, Hungary, Iran, Israel, Italy, Japan, Kyrgyzstan, Latvia, Lithuania, Mongolia, Morocco (Doukale Daief et al. 2025), Netherlands, Poland, Portugal, Romania, Russia, Slovakia, Spain, Sweden, Switzerland, Syria, Tajikistan, The United Kingdom, Tunisia (Soós and Papp 1984), Turkey (Dursun et al. 2015), Turkmenistan, Ukraine, Uzbekistan; Oriental: China (Taiwan), India; Afrotropical: South Africa.

**20. Cerodontha (Icteromyza) nigricoxa (Malloch, 1914) (as *Agromyza*)**

**Literature.**Spencer 1961, 1962.

**Distribution.** Oriental: China (Taiwan).

**21. Cerodontha (Icteromyza) piliseta (Becker, 1903) (as *Agromyza*)**

*Agromyza flavofemorata* Malloch, 1914 (synonym).

*Agromyza pubicornis* Lamb, 1912 (synonym).

Cerodontha (Icteromyza) hirsuta Sasakawa, 1972 (synonym).

**Literature.**Spencer 1959, 1961, 1986; Sasakawa 1972, 2008, 2013; Zlobin 2000.

**Distribution.** Palaearctic: Egypt, Greece, Spain; Oriental: China (Taiwan), Sri Lanka, Thailand; Australian/Oceanian: Australia, Fiji, Guam, Melanesia, Micronesia, Papua New Guinea, Polynesia, Samoa, Solomon Islands, Vanuatu; Afrotropical: Cabo Verde, Kenya (https://www.gbif.org/species/1552609), Seychelles, Zimbabwe.

**22. Cerodontha (Icteromyza) rishii Garg, 1971**

**Literature.**Garg 1971; Spencer 1986; Zlobin 2000.

**Distribution.** Oriental: China (Fujian, Taiwan, Yunnan), India, Indonesia, Nepal, Philippines, Thailand.

**23. Cerodontha (Icteromyza) taipingensis (Shiao & Wu, 1997) (as *Metopomyza*)**

**Literature.**Shiao and Wu 1997 (1996).

**Distribution.** Oriental: China (Taiwan).

**24. Cerodontha (Poemyza) beigerae Nowakowski, 1972**

**Literature.**Nowakowski 1972, 1973; Zlobin 1993a; Černý 2007; Černý and van Zuijlen 2022.

**Distribution.** Palaearctic: Belgium, China (Inner Mongolia)^#^, Czech Republic, Germany, Hungary, Mongolia, Netherlands, North Korea, Poland, Russia, Slovakia, Sweden, Switzerland.

**25. Cerodontha (Poemyza) bisetiorbita (Sasakawa, 1955) (as *Phytobia*)**

**Literature.**Sasakawa 1955, 1961, 2005.

**Distribution.** Palaearctic: Japan (Honshu); Oriental: China (Taiwan).

**26. Cerodontha (Poemyza) hirta Sasakawa, 1972**

*Cerodontha hirtipennis* Sasakawa, 1977 (synonym).

**Literature.**Sasakawa 1972; Sasakawa and Fan 1985; Zlobin 2001b.

**Distribution.** Oriental: China (Taiwan), Myanmar.

**27. Cerodontha (Poemyza) incisa (Meigen, 1830) (as *Agromyza*)**

*Agromyza carbonella* Zetterstedt, 1860 (synonym).

*Agromyza graminis* Kaltenbach, 1873 (synonym).

*Oscinis okazakii* Matsumura, 1916 (synonym).

**Literature.**Spencer1976; Nowakowski 1973; Soós and Papp 1984; Spencer 1990; Lonsdale 2021.

**Distribution.** Palaearctic: Andorra, Armenia, Austria, Azerbaijan, Belarus, Bulgaria, Croatia, Czech Republic, Estonia, Finland, France, Georgia, Germany, Hungary, Iceland, Ireland, Italy, Japan, Kyrgyzstan, Latvia, Lithuania, Mongolia, Netherlands, Norway, Pakistan, Poland, Portugal, Romania, Russia, Slovakia, Spain, Sweden, Switzerland, Tajikistan, The United Kingdom, Turkey, Ukraine, Uzbekistan (Soós and Papp 1984); Oriental: China (Fujian); Nearctic: Canada, USA; Afrotropical: Madagascar (https://www.gbif.org/species/4571482).

**28. Cerodontha (Poemyza) lateralis (Macquart, 1835) (as *Agromyza*)**

*Agromyza vittigera* Zetterstedt, 1848 (synonym).

*Agromyza variceps* Zetterstedt, 1860 (synonym).

*Agromyza laminata* Brischke, 1881 (synonym).

**Literature.**Nowakowski 1973; Spencer 1976; Soós and Papp 1984; Zlobin 2005, Černý 2018.

**Distribution.** Palaearctic: Afghanistan, Armenia, Azerbaijan, Belarus, Bulgaria, China (Xinjiang), Croatia, Czech Republic, Denmark, Estonia, Finland, France, Germany, Greece, Hungary, Iran, Italy, Japan, Kazakhstan, Kyrgyzstan, Latvia, Lithuania, Moldova, Mongolia, Netherlands, Norway, Poland, Russia, Serbia, Spain, Sweden, Switzerland, Tajikistan, The United Kingdom, Tunisia, Turkey, Turkmenistan, Ukraine, Uzbekistan (Soós and Papp 1984); Nearctic: Canada, USA; Afrotropical: Madagascar (https://www.gbif.org/species/4571482).

**29. Cerodontha (Poemyza) muscina (Meigen, 1830) (as *Agromyza*)**

*Agromyza marginata* Loew, 1869 (synonym).

**Literature.**Spencer 1976; Soós and Papp 1984; Dursun et al. 2015; Černý et al. 2018; Guglya 2021; Lonsdale 2021; Černý et al. 2022.

**Distribution.** Palaearctic: Andorra, Austria, Belarus, Belgium, Bulgaria, China (Inner Mongolia)^#^, Croatia, Czech Republic, Denmark, Estonia, Finland, France, Germany, Greece, Hungary, Italy, Kyrgyzstan, Latvia, Lithuania, Netherlands, Norway, Poland, Portugal, Romania, Russia, Slovakia, South Korea, Spain, Sweden, Switzerland, The United Kingdom, Ukraine (Soós and Papp 1984); Nearctic: Canada, USA; Afrotropical: Madagascar (https://www.gbif.org/species/1552471).

**30. Cerodontha (Poemyza) oryziphila Zlobin, 1993**

**Literature.**Zlobin 1993a, Zlobin 2001b.

**Distribution.** Oriental: China (Guangdong), Malaysia.

**31. Cerodontha (Poemyza) setariae (Spencer, 1959) (as *Phytobia*)**

**Literature.**Spencer 1959, 1990; Sasakawa 1961.

**Distribution.** Palaearctic: China (Shanghai); Japan; Afrotropical: Sierra Leone.

**32. Cerodontha (Poemyza) superciliosa (Zetterstedt, 1860) (as *Agromyza*)**

*Agromyza coquilletti* Malloch, 1913 (synonym).

**Literature.**Nowakowski 1973; Spencer 1976; Zlobin 2005; Karpa 2007; Lonsdale 2021.

**Distribution.** Palaearctic: China (Xinjiang), Czech Republic, Denmark, Finland, France, Germany, Hungary, Italy, Kazakhstan, Latvia, Lithuania, Netherlands, Poland, Russia, Slovakia, Sweden, Spain, Switzerland, The United Kingdom, Turkmenistan, Ukraine, Uzbekistan; Nearctic: Canada, USA.

## Supplementary Material

XML Treatment for Cerodontha (Cerodontha) flavicornis

XML Treatment for Cerodontha (Cerodontha) fulvipes

XML Treatment for Cerodontha (Dizygomyza) flavilunulata

XML Treatment for Cerodontha (Dizygomyza) granditerga

XML Treatment for Cerodontha (Dizygomyza) labradorensis

XML Treatment for Cerodontha (Dizygomyza) tumefacta

XML Treatment for Cerodontha (Icteromyza) geniculata

XML Treatment for Cerodontha (Poemyza) beigerae

XML Treatment for Cerodontha (Poemyza) muscina

## ﻿Additional information

### Use of AI

No use of AI was reported.

### Author ORCIDs

Xin-Ting Fu https://orcid.org/0009-0002-3970-8367

Li Shi https://orcid.org/0000-0001-5842-4904

Shi-Mingzhe Wang https://orcid.org/0009-0004-8689-1816

### Data availability

All of the data that support the findings of this study are available in the main text.
